# Spatiotemporal profiles of gene activity in stamen delineate nucleo-cytoplasmic interaction in a male-sterile somatic cybrid citrus

**DOI:** 10.1093/hr/uhad105

**Published:** 2023-05-12

**Authors:** Nan Jiang, Meng-Qi Feng, Lai-Chao Cheng, Li-Hua Kuang, Chao-Chao Li, Zhao-Ping Yin, Rong Wang, Kai-Dong Xie, Wen-Wu Guo, Xiao-Meng Wu

**Affiliations:** National Key Laboratory for Germplasm Innovation & Utilization of Horticultural Crops, College of Horticulture and Forestry Sciences, Huazhong Agricultural University, Wuhan, 430070, China; National Key Laboratory for Germplasm Innovation & Utilization of Horticultural Crops, College of Horticulture and Forestry Sciences, Huazhong Agricultural University, Wuhan, 430070, China; National Key Laboratory for Germplasm Innovation & Utilization of Horticultural Crops, College of Horticulture and Forestry Sciences, Huazhong Agricultural University, Wuhan, 430070, China; National Key Laboratory for Germplasm Innovation & Utilization of Horticultural Crops, College of Horticulture and Forestry Sciences, Huazhong Agricultural University, Wuhan, 430070, China; National Key Laboratory for Germplasm Innovation & Utilization of Horticultural Crops, College of Horticulture and Forestry Sciences, Huazhong Agricultural University, Wuhan, 430070, China; National Key Laboratory for Germplasm Innovation & Utilization of Horticultural Crops, College of Horticulture and Forestry Sciences, Huazhong Agricultural University, Wuhan, 430070, China; National Key Laboratory for Germplasm Innovation & Utilization of Horticultural Crops, College of Horticulture and Forestry Sciences, Huazhong Agricultural University, Wuhan, 430070, China; National Key Laboratory for Germplasm Innovation & Utilization of Horticultural Crops, College of Horticulture and Forestry Sciences, Huazhong Agricultural University, Wuhan, 430070, China; National Key Laboratory for Germplasm Innovation & Utilization of Horticultural Crops, College of Horticulture and Forestry Sciences, Huazhong Agricultural University, Wuhan, 430070, China; Hubei Hongshan Laboratory, Wuhan 430070, China; National Key Laboratory for Germplasm Innovation & Utilization of Horticultural Crops, College of Horticulture and Forestry Sciences, Huazhong Agricultural University, Wuhan, 430070, China

## Abstract

Cytoplasmic male sterility (CMS) has long been used to produce seedless fruits in perennial woody crops like citrus. A male-sterile somatic cybrid citrus (G1 + HBP) was generated by protoplast fusion between a CMS callus parent ‘Guoqing No. 1’ Satsuma mandarin (*Citrus unshiu*, G1) and a fertile mesophyll parent Hirado Buntan pummelo (*Citrus grandis*, HBP). To uncover the male-sterile mechanism of G1 + HBP, we compared the transcriptome profiles of stamen organ and cell types at five stages between G1 + HBP and HBP, including the initial stamen primordia, enlarged stamen primordia, pollen mother cells, tetrads, and microspores captured by laser microdissection. The stamen organ and cell types showed distinct gene expression profiles. A majority of genes involved in stamen development were differentially expressed, especially *CgAP3.2*, which was downregulated in enlarged stamen primordia and upregulated in tetrads of G1 + HBP compared with HBP. Jasmonic acid- and auxin-related biological processes were enriched among the differentially expressed genes of stamen primordia, and the content of jasmonic acid biosynthesis metabolites was higher in flower buds and anthers of G1 + HBP. In contrast, the content of auxin biosynthesis metabolites was lower in G1 + HBP. The mitochondrial tricarboxylic acid cycle and oxidative phosphorylation processes were enriched among the differentially expressed genes in stamen primordia, meiocytes, and microspores, indicating the dysfunction of mitochondria in stamen organ and cell types of G1 + HBP. Taken together, the results indicate that malfunction of mitochondria-nuclear interaction might cause disorder in stamen development, and thus lead to male sterility in the citrus cybrid.

## Introduction

Cytoplasmic male sterility (CMS), a maternally inherited trait determined by mitochondrial genomes, confers a typical non-functional male gametocyte in flowering plants. CMS has long been used for crossbreeding and hybrid seed production in crops, for its benefit of avoiding laborious manual emasculation [1–3]. In fruit crops like citrus, CMS has been utilized to produce seedless fruits, which is a desirable trait for the fresh market. CMS is usually caused by the mitochondrial chimeric open reading frames (ORFs) that co-transcribe with the conserved mitochondrial genes, which encode proteins that disturb mitochondrial reactions like ATP synthesis and oxygen respiration [4, 5]. The CMS-causal chimeric ORFs have been identified in rice [6, 7], oilseed rape [8], and tomato [9, 10].

CMS is a result of nuclear–mitochondrial interaction, including mitochondrial retrograde regulation (MRR) and mitochondrial anterograde regulation (MAR). In the MRR mode, mitochondrial signals like ROS, Ca^2+^ and ATP together with hormones affect the expression of nuclear genes [11, 12]. The nuclear genes that change expression in CMS plants are those related to programmed cell death, hormone signal transduction and flower organ development [13, 14]. The anther development-related genes showed delayed or extended expression in *Brassica* CMS lines with abnormal flower development, such as *bHLH89*, *bHLH91*, *MYB80/MS188*, *MYB35/TDF1*, and *AMS* (*ABORTED MICROSPRES*) [15, 16]. In the MAR mode, nucleus-to-mitochondria signaling contributes to the regulation of mitochondrial functions [17]. Nuclear restorer-of-fertility (Rf) genes splice, edit and/or cleave mitochondrial chimeric ORFs at post-transcriptional level to restore male fertility in CMS lines [18]. Rf genes were identified in CMS lines, including rice [19], oilseed [20], radish [21, 22], and pepper [23].

CMS is artificially classified into two types according to the origin of the CMS-causal genes. Autoplasmy CMS arises from spontaneous mutation in the mitochondrial genome, whereas alloplasmy CMS is a result of nuclear–mitochondrial incompatibility following cytoplasmic substitutions [24]. Somatic hybridization is one approach to producing alloplasmy CMS lines. In citrus, protoplast fusion allows the production of novel germplasm and circumvents the difficulties in traditional citrus breeding, like sexual incompatibility, polyembryony, and male or female sterility [25]. Generation of somatic cybrids with the nuclear genome from a seedy parent and the cytoplasmic genomes from a CMS parent provides an effective approach for seedless improvement in citrus [26, 27].

Seedlessness is a desirable trait for citrus breeding, and male sterility is the main cause of seedlessness in the most widely cultivated citrus varieties, including Satsuma mandarin (*Citrus unshiu*). Male sterility in Satsuma mandarin has been supposed to be CMS according to the segregation ratios of reciprocal crosses [28, 29]. In our previous breeding program, a male-sterile somatic cybrid pummelo (G1 + HBP) was regenerated from somatic fusion between the callus protoplast of CMS ‘Guoqing No. 1’ Satsuma mandarin (G1) and the mesophyll protoplast of fertile Hirado Buntan pummelo (HBP) [30]. Molecular and omics data demonstrated that the mitochondrial genome of G1 + HBP is from the CMS callus parent G1, while the nuclear and chloroplast genomes of G1 + HBP are from the fertile mesophyll parent HBP [30–32]. Cytological observation revealed defects of stamen development in G1 + HBP, and the nuclear genes for male sterility were identified by comparative transcriptome and proteome analysis in the floral buds between G1 + HBP and HBP, such as the floral organ development-related ABC genes *AP3* and *PI*, as well as *CYP716* and *GA20ox* [33, 34]. According to small RNA high-throughput sequencing, the differentially expressed miR167 and miR399 were identified to be the candidate genes involved in male sterility of G1 + HBP [35, 36], and the function of the miR399-*CsUBC24* module in male sterility was validated in the short juvenile mini-citrus (*Fortunella hindsii*) [37]. According to the pan-mitogenomes of citrus, we revealed the genetic basis of cytonuclear conflicts in citrus cybrids including G1 + HBP, and identified two CMS-related chimeric mitochondrial ORFs [38]. However, the mechanism of CMS in the citrus cybrids is still to be elucidated. In this study we conducted a spatiotemporal comparison of transcriptomic profiles in laser-captured stamen primordia, meiocytes, and microspores between the somatic cybrid G1 + HBP and its mesophyll parent HBP. The distinct mitochondrial regulation of stamen development was investigated, with emphasis on the influence of dysfunctional mitochondria on stamen development-related genes, and thus we propose reliable clues about the male-sterile mechanism in the somatic cybrid citrus.

## Results

### Male-sterile morphology and flower development deficiency in G1 + HBP

**Figure 1:**
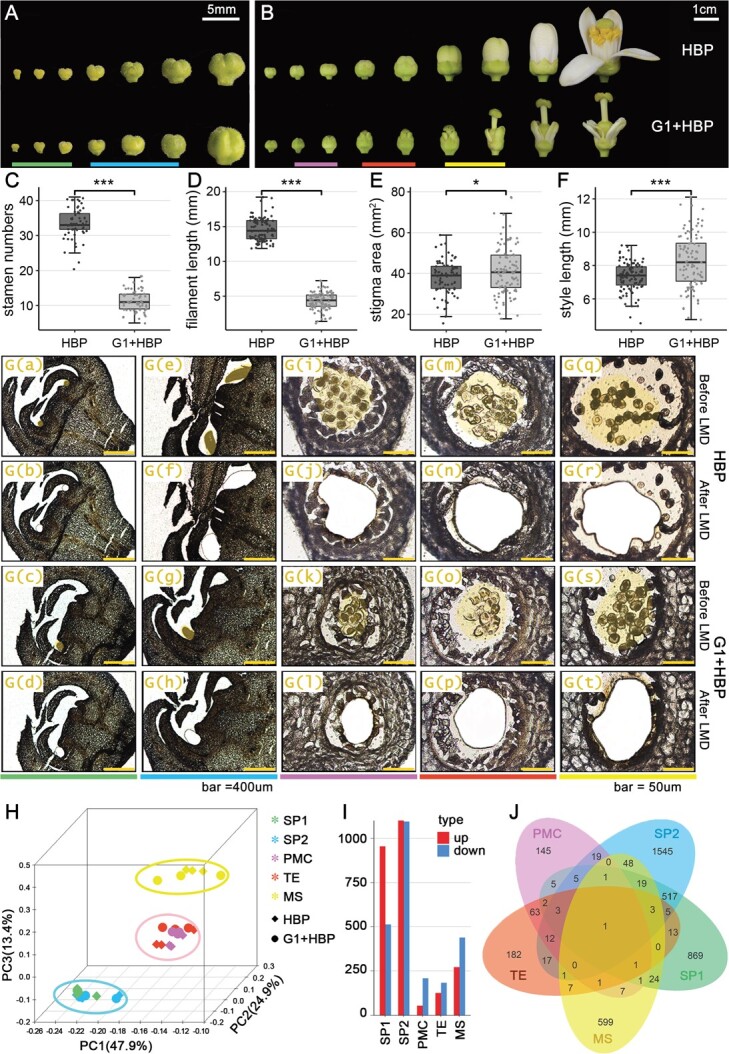
Morphological observations of flower development and isolation of stamen organ and cell types in HBP and G1 + HBP using laser microdissection. (A, B) Entire process of flower development in male-sterile somatic cybrid pummelo (G1 + HBP) and its fertile mesophyll parent Hirado Buntan pummelo (*Citrus grandis*, HBP), including stamen primordia, and meiocyte and microspore development stages. Scale bars: (A) = 5 mm; (B) = 1 cm. (C) Stamen numbers. (D) Filament length. (E) Stigma area. (F) Style length. In (C), *n* = 60. In (D–F), *n* = 100. In (C–F), the horizontal line in the box represents the median, the box limits represent upper and lower quartiles, and the points represent individuals. ^*^P < .05, ^***^P < .001 (Student’s *t*-test). (G) (a–t) Isolation of stamen organ and cell types by LMD. Yellow shadows indicate five stamen organ and cell types. Scale bars = 400 μm in stamen primordia (a–h) and 50 μm in meiocytes (i–p) and microspores (q–t). (H) PCA of unigenes in stamen organ and cell types of HBP and G1 + HBP. The first three principal components are shown. (I) Statistics of upregulated/downregulated DEGs in stamen organ and cell types of G1 + HBP compared with HBP. (J) Venn diagram showing the intersection of DEGs in stamen organ and cell types of G1 + HBP compared with HBP. In (D) and (E), DEGs are identified using DEseq2 with *q* value <0.05. Colored lines at the bottom in (A), (B) and (G) (a–t) correspond to stamen organ and cell types in H and I. Spring green, initiated stamen primordia (SP1); sky blue, enlarged stamen primordia (SP2); orchid, pollen mother cell (PMC); orange-red, tetrads (TE); yellow, microspores (MS).

The floral development process of HBP and G1 + HBP was investigated in morphologically and cytologically (Fig. 1 A and B, Supplementary Data Fig. S1A–T). The flower organs, especially the stamens and petals, differ in G1 + HBP from HBP. The space between petal and carpel in G1 + HBP was narrower than that in HBP, bearing significantly fewer stamens, as well as fewer stamen primordia at both initial and development stages (Fig. 1C, Supplementary Data Fig. S1A and I–L). After anther formation, more obvious stamen defects emerged in G1 + HBP compared with HBP, including smaller anthers, shorter filament, blurry junction between anther and filament, and fusion between stamens and petals/carpels (Fig. 1D, Supplementary Data Fig. S1F and G). In G1 + HBP, only a few anthers formed smaller pollen sacs and disordered anther walls, with few microspores and no dehiscence compared with HBP (Supplementary Data Fig. S1H and M–T). Petals in G1 + HBP were curved and developed into small and narrow mature petals in the white petal stage (Supplementary Data Fig. S1B and C). Degenerated petals might cause premature exposure of the stigma in G1 + HBP, which resulted in bruising and splitting of the stigma (Supplementary Data Fig. S1D). The style length and stigma area varied in G1 + HBP, while the average style length and stigma area were both increased in G1 + HBP compared with HBP (Fig. 1E and F). Taking these results together, we observed male sterility and flower malformation in the citrus somatic cybrid G1 + HBP.

To profile the gene expression differences in the stamen between G1 + HBP and HBP, five stamen development stages were selected for LMD capturing of stamen organ and cell types (Fig. 1A, B, and G). The initial stamen primordium (SP1) was captured from young flower buds 1–2 mm in length. The enlarged stamen primordium (SP2) was captured from flower buds of 2–4 mm. Anthers formed later, in flower buds of >5 mm (Fig. 1B). Different gamete cell types were captured by laser from the developing anther chamber, including pollen mother cells (PMCs) when the petals exposed from the sepals, tetrad cells (TEs) when the sepals were one-third to one-half of petal length, and microspores (MSs) when the sepals were shorter than half the length of the petals (Fig. 1A, B, and G).

### Gene expression profiles in stamen organ and cell types of HBP and G1 + HBP

A total of area of stamen organ and cell types of 300–500 mm^2^ was collected from each sample for RNA isolation and RNA-seq (Supplementary Data Table S2, Supplementary Data Fig. S2). Of the clean reads in each sample, 72–96% were mapped to the pummelo reference genome (Supplementary Data Table S3). A total of 20 784 assembled unigenes expressed in at least one tissue were annotated (Supplementary Data Set S1, Supplementary Data Fig. S3A and B). Among these unigenes, only 47% (9711) were expressed in all tissues (Supplementary Data Fig. S3A). Pearson correlation and PCA showed high correlation of biological replications. The samples were clustered into three discrete groups, including group 1 of stamen primordia (SP1, SP2), group 2 of meiocytes (PMC, TE) and group 3 of microspores (MS) (Fig. 1H, Supplementary Data Fig. S3C).

A total of 4114 DEGs were identified in stamen organ and cell types of G1 + HBP compared with HBP (Fig. 1I and J, Supplementary Data Set S2). The number of DEGs in the stamen primordia (SP1, SP2) was much greater than that in the meiocytes (PMC, TE) and the microspores (MS) (Fig. 1I). Upregulated DEGs in the stamen primordia (SP1, SP2) were more numerous than downregulated DEGs, while downregulated DEGs in the meiocytes (PMC, TE) and microspores (MS) were more than numerous than upregulated DEGs (Fig. 1I). The majority of DEGs were specific to individual stamen organ and cell types, while DEGs of stamen organ and cell types within the same group had more overlaps (Fig. 1J).

According to the comparison of our LMD transcriptome data with the previous transcriptome data of flower buds in HBP and G1 + HBP, we identified subsets of genes (7294) specifically expressed in stamen organ and cell types (Supplementary Data Sets S3 and S4). The expression abundance of these specifically expressed genes was lower than that of the whole set of expressed genes in stamen organ and cell types, and more specifically expressed genes were identified in group 2 (PMC, TE) and group 3 (MS) cells compared with group 1 (SP1, SP2) organs (Supplementary Data Fig. S3D and E).

Weighted gene co-expression network analysis (WGCNA) was conducted in 17 642 unigenes with a high coefficient of expression variation (CV > 0.5) across all LMD samples. The genes with a weighted correlated expression pattern across all samples were clustered in a module, with 42 co-expression modules generated (Supplementary Data Fig. S4A and B). These modules were classified into three groups, which were highly correlated with the group 1 (SP1, SP2) organs and group 2 (PMC, TF) and group 3 (MS) cell types of G1 + HBP and HBP according to the distribution of hierarchical clusters. The exception was the dark orange module, which was manually moved to group 2 (Supplementary Data Fig. S4B). Ten modules with high correlation (>.9) with the stamen organ and cell types were identified as tissue-specific expression modules (Supplementary Data Figs S4B and C and S5A). A total of 18 transcription factors families including 496 transcription factors were enriched among the 10 modules, based on which the weighted networks were constructed (Supplementary Data Figs S4C and S5B and C).

In summary, the global transcriptome profiles and distribution of DEGs suggest that stamen primordia (SP1, SP2), meiocytes (PMC, TE), and microspores (MS) have distinct gene expression profiles corresponding to the development stages. Abnormal gene expression persists from stamen primordia initiation to meiosis and microspore formation in cybrid G1 + HBP compared with HBP. However, the more drastic gene expression abnormality in the stamen primordium suggests that the male sterility of G1 + HBP starts from deficient stamen primordium initiation followed by abnormal pollen development.

### Expression patterns of differentially expressed genes in stamen organ and cell types of HBP and G1 + HBP

**Figure 2:**
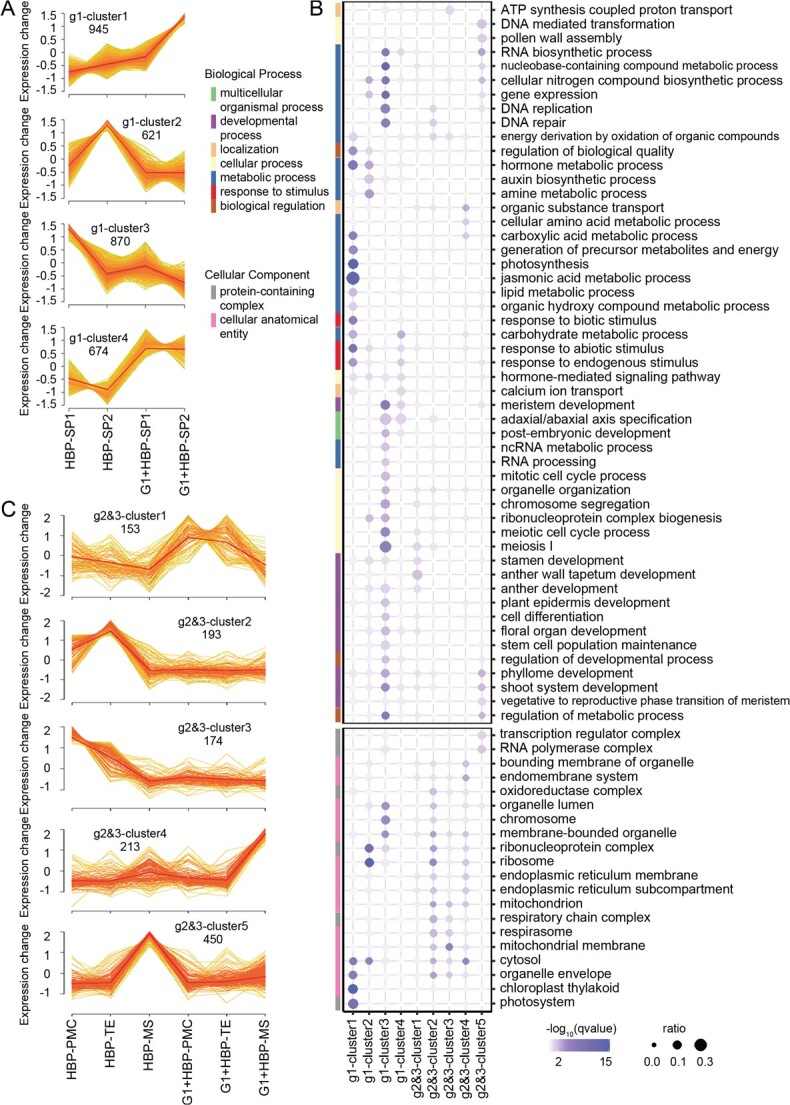
Expression pattern and GO enrichment of DEGs in grouped stamen organ and cell types of HBP and G1 + HBP. (A, C) Expression pattern of DEGs in grouped samples. Normalized TPM is used for c-means soft clustering with Mfuzz; the *y*-axis indicates normalized TPM using *z*-score. In (A), DEGs in group 1 organs (SP1 and SP2) were grouped into four clusters based on their expression level. In (C), DEGs in group 2 (PMC and TE) and group 3 (MS) cell types were grouped into five clusters based on their expression level. (B) GO enrichment of DEGs with *q* value <.05 as significant, with the biological processes and cellular components exhibited. The *x*-axis indicates clusters, the *y*-axis indicates enriched GO items, size indicates percentage of identified genes in the background (scored as gene ratio), color indicates significance level [scored as −log_10_ (*q* value)] using Benjamini–Hochberg correction, and line color indicates second levels of GO items.

A total of 3110 DEGs were identified in group 1 (SP1, SP2) organs, generating four expression patterns (g1-clusters 1–4). Expression of DEGs in g1-cluster 1 increased from SP1 to SP2 in both HBP and G1 + HBP, but their expression level was much higher in G1 + HBP than in HBP (Fig. 2A). The JA metabolic process, photosynthesis, and chloroplast thylakoid component were enriched among DEGs in g1-cluster 1 (Fig. 2B). Expression of DEGs in g1-cluster 2 drastically increased from SP1 to SP2 in HBP but was stably low in G1 + HBP (Fig. 2A). Auxin biosynthesis process and ribosome component were enriched among DEGs in g1-cluster 2 (Fig. 2B). Expression of DEGs in g1-cluster 3 decreased from SP1 to SP2 in both HBP and G1 + HBP, but the expression level was much lower in G1 + HBP than in HBP (Fig. 2A). Development-related biological processes and nuclear metabolic processes were enriched among DEGs in g1-cluster 3 (Fig. 2B). Expression of DEGs in g1-cluster 4 were decreased from SP1 to SP2 in HBP, but the expression levels were higher in G1 + HBP than in HBP (Fig. 2A). The enriched GO terms in g1-cluster 4 coincided with those in g1-cluster 1 and g1-cluster 3, mainly including hormone and development-related processes (Fig. 2B). In summary, DEGs in group 1 (SP1, SP2) organs were associated with many biological process changes, including JA metabolic and photosynthesis process among upregulated genes in G1 + HBP compared with HBP (Fig. 2, Supplementary Data Set S5). Among the downregulated genes in group 1 (SP1, SP2) organs of G1 + HBP compared with HBP, there were stamen formation- and development-related genes *SUPERMAN* (*CgSUP*) and UNUSUAL FLORAL ORGANS (*CgUFO*), anther cell differentiation- and development-related genes *SPOROCYTELESS/NOZZLE* (*CgSPL/NZZ*) and *EXCESS MICROSPOROCYTES 1* (*CgEMS1*), anther patterning and dehiscence related CC-type floral glutaredoxins *CgROXY2*, bZIP transcription factor *CgTGA9* and *CgTGA10*, and stem cell maintenance genes *SHOOT MERISTEMLESS* (*CgSTM*) and *WUSCHEL* (*CgWUS*) (Supplementary Data Set S6).

A total of 1183 DEGs were identified in group 2 (PMC, TE) and group 3 (MS) cells, forming five expression patterns (g2- and 3-clusters 1–5) (Fig. 2C). Expression of DEGs in g2- and 3-cluster 1 moderately decreased from PMC to MS in both HBP and G1 + HBP, but the expression level of the entire DEGs was higher in G1 + HBP than in HBP (Fig. 2C). Stamen development-related processes were enriched among DEGs in g2- and 3-cluster 1 (Fig. 2B). Expression of DEGs in g2- and 3-cluster 2 increased from PMC to TE and then decreased in MS of HBP, while expression of DEGs in g2- and 3-cluster 3 continued to decrease from PMC to TE and MS of HBP (Fig. 2C). However, expression of these DEGs remained unchanged from PMC to MS in G1 + HBP, and the expression level of these DEGs was lower in group 2 (PMC, TE) cells of G1 + HBP than in HBP (Fig. 2C). Similar cellular components, including mitochondria and respirasomes, were enriched among DEGs in g2- and 3-cluster 2 and g2- and 3-cluster 3. In addition, cellular respiration process was enriched among DEGs in g2- and 3-cluster 2, and ATP synthesis-coupled proton transport was enriched among DEGs in g2- and 3-cluster 3 (Fig. 2B). Expression of DEGs in g2- and 3-cluster 4 remained stable in PMC and TE, and increased drastically in MS of G1 + HBP, but remained stable from PMC to MS in HBP (Fig. 2C). Localization, transport, mitochondria, vesicle, and endoplasmic reticulum were enriched among these DEGs (Fig. 2B, Supplementary Data Set S5). Expression of DEGs in g2- and 3-cluster 5 remained stable in PMC and TE but increased drastically in MS of HBP, but remained stable from PMC to MS in G1 + HBP (Fig. 2C). Mitochondrial DNA metabolic process and pollen wall assembly were enriched among these DEGs (Fig. 2B). In summary, DEGs in group 2 (PMC, TE) cells were associated with many cellular component changes, especially mitochondria, as well as energy metabolism-related process. Significantly, these DEGs were downregulated in G1 + HBP. DEGs in group 3 (MS) cells were associated with metabolism and transport, and they were upregulated in G1 + HBP (Fig. 2, Supplementary Data Set S5). Many meiosis-related genes showed higher expression in group 2 (PMC, TE) cells of G1 + HBP compared with HBP, including synaptonemal complex-related genes *CgASY1* and *CgZIP1*, double strand break formation-related genes *CgPRD3*, *CgDFO*, and *CgMTOPVIB* (Supplementary Data Set S6). The tapetum and pollen development-related genes *DYSFUNCTIONAL TAPETUM 1* (*CgDYT1*) and *CgTDF1* were upregulated in PMC, while the sporopollenin biosynthesis-related genes *ABORTED MICROSPORES* (*CgAMS*), *CgCYP703A2*, *CgCYP704B1*, *MALE STERILE 2* (*CgMS2*), and *POLYKETIDE SYNTHASE 6* (*CgLAP6*) were downregulated in MS of G1 + HBP compared with HBP (Supplementary Data Set S6).

The total 4114 DEGs in stamen organ and cell types were clustered in G1 + HBP and HBP respectively. Among them, 70% (1982/2832) with membership >0.5 shared similar expression patterns and were grouped into four clusters (clusters 1–4) according to expression patterns, while the remaining 2132 DEGs were designated as genes with differential expression patterns (DEPGs) (Supplementary Data Fig. S6A–B). These clusters also showed a distinct distribution of GO terms in stamen organ and cell types. Stamen and flower development process, photosynthesis, and JA metabolic process were highly associated with stamen primordia; cellular respiration and meiotic cell cycle were highly associated with meiocytes; and nucleobase metabolic process was highly associated with microspores (Supplementary Data Fig. S6C). In summary, the DEGs with similar expression pattern between G1 + HBP and HBP in each cluster indicated the disruption of essential processes required for stamen organ development and cell type formation in G1 + HBP. In addition, the GSEA results also demonstrated the importance of JA and IAA metabolism, oxidative phosphorylation (OXPHOS) and tricarboxylic acid cycle (TCA) pathways in stamen organ and cell types (Supplementary Data Fig S7).

### Expression of ABCE model genes in stamen organ and cell types of HBP and G1 + HBP

**Figure 3:**
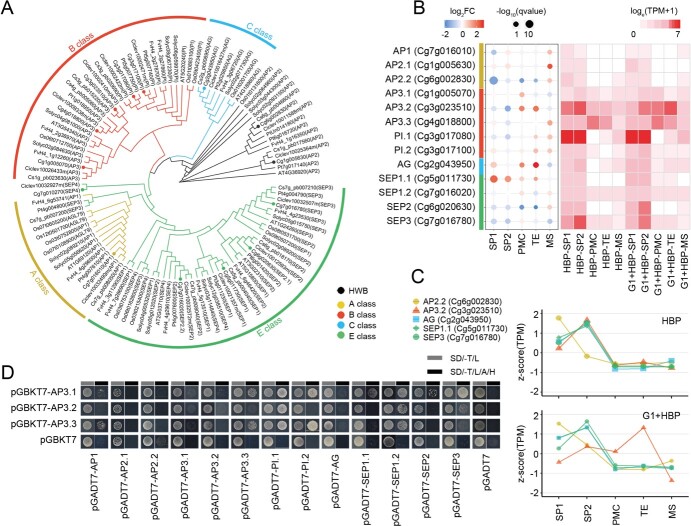
ABCE model genes in cybrid pummelo G1 + HBP. (A) Phylogenetic analysis of ABCE model genes in citrus (*C. sinensis*, *C. grandis*, *C. clementina*) and *P. trifoliata*, with *A. thaliana*, *S. lycopersicum*, *F. vesca*, and *O. sativa*. The pummelo banches are marked with dots. (B) Expression analysis of identified ABCE model homologous genes in stamen organ and cell types of HBP and G1 + HBP. Circle color represents fold change (scored as log_2_ fold change), circle size represents significance of differential expression [scored as −log_10_ (*q* value)]. Color in the heat map represents expression value [scored as log_e_ (TPM + 1)]. (C) Expression pattern of differentially expressed ABCE model genes in stamen organ and cell types of HBP and G1 + HBP. The shapes of the nodes in each line represent individual genes and the *y* axis indicates the normalized TPM using the *z*-score. In (A–C) the color legend indicates classes of ABCE model genes: yellow, red, sky blue, and spring green represent A, B, C, E class genes, respectively. (D) Y2H assay. *CgAP3* was cloned into pGBKT7 as the bait and ABCE model genes were cloned into pGADT7 as preys. Interactions between *CgAP3* and ABCE model genes were detected by co-transformation of a pair of bait and prey vectors into a yeast strain. Transformants were spotted on SD medium without Trp and Leu (SD/−T/L, labeled gray) or SD medium without Trp, Leu, Ade, and His (SD/−T/L/A/H, labeled black).

ABCE model genes play a vital role in flower organ identity and development. ABCE homologous genes were identified in the genera *Citrus* (*C. sinensis*, *C. grandis*, *C. clementina*) and *Poncirus* (*P. trifoliata*). The phylogenetic analysis of ABCE model genes in citrus was conducted with *Arabidopsis*, rice, tomato, and strawberry (Fig. 3A). Interestingly, multiple members were identified in B-class genes of citrus, such as two *PISTILLATA* (*PI*) and three *APETALA 3* (*AP3*) in *Citrus*, in contrast to the sole member in *Arabidopsis* and rice (Fig. 3A).

Almost all the ABCE genes were detectable in the LMD transcriptome. Most of these genes were expressed at a high level in stamen primordia, especially the B-class genes (Fig. 3B). The three members of *CgAP3* showed different expression patterns. In HBP, *CgAP3.1* and *CgAP3.2* were expressed at a higher level in group 1 (SP1, SP2) organs, while *CgAP3.3* was expressed at a higher level in group 2 (PMC, TE) cells (Fig. 3B). The expression pattern of *CgAP3* was also different in flower buds and anthers of HBP and G1 + HBP. *CgAP3.1* expression was higher in anthers (MEA and MSA) than in flower buds (SPF), and upregulated in MSA of G1 + HBP compared with HBP. *CgAP3.2* expression was consistent in SPF, MEA, and MSA, and upregulated in SPF of G1 + HBP compared with HBP. *CgAP3.3* expression was higher in flower buds (SPF) than in anthers (MEA, MSA), and upregulated in SPF and downregulated in MEA of G1 + HBP compared with HBP (Supplementary Data Fig. S8A). Five ABCE model DEGs were identified, covering each class of ABCE model genes (Fig. 3B and C). Most of these genes were differentially expressed in group 1 (SP1, SP2) organs of G1 + HBP and HBP, and *CgAP3.2* and *CgAG (AGAMOUS)* were also upregulated in TE of G1 + HBP (Fig. 3B and C). Notably, only *CgAP3.2* was a DEPG downregulated in SP2 but upregulated in TE of G1 + HBP compared with HBP (Fig. 3B and C).

The phylogenetic analysis demonstrated that CgAP3.1 was a member of the TM6 lineage, which is absent in *Arabidopsis* and rice, while CgAP3.2 and CgAP3.3 were core eudicot AP3 genes with an independent *Citrus* genus branch (Supplementary Data Fig. S8B). All three CgAP3s interacted with CgPI and CgSEP1.2 in the yeast two hybridization (Y2H) assay (Fig. 3D), and homologous interactions have also been validated in other plant species [39, 40]. In addition, the three CgAP3s in citrus had unique interaction patterns. CgAP3.1 interacted with CgSEP1.1 and CgSEP2 and CgAP3.1 and CgAP3.2 interacted with CgSEP3, while CgAP3.2 only interacted with CgPI.1 but not CgPI.2 (Fig. 3D).

### Change of jasmonic acid and auxin metabolic and signaling pathways in G1 + HBP

**Figure 4:**
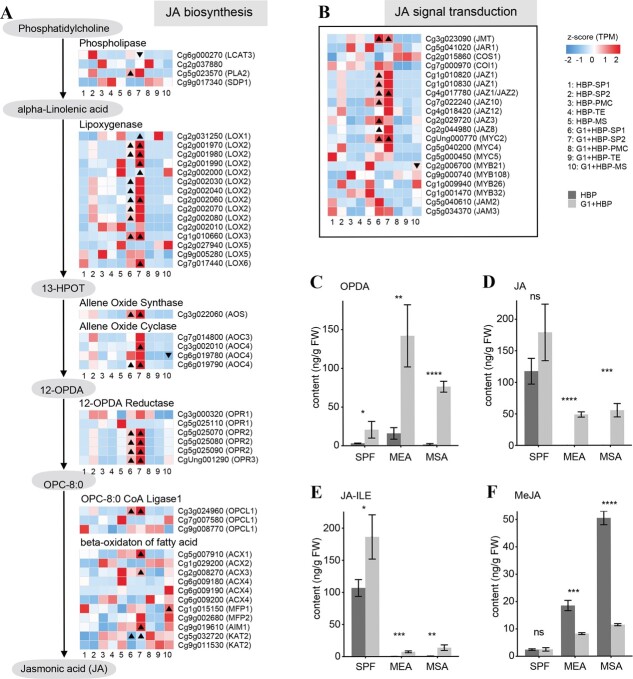
Expression level of JA pathway genes and content of jasmonates in stamens of HBP and G1 + HBP. Expression level of JA biosynthesis pathway (A) genes and JA signal transduction (B) genes in stamen organ and cell types of HBP and G1 + HBP. In (A) and (B), color in the heat map indicates normalized TPM by *z*-score, DEGs are marked with triangles, regular triangles indicate significantly upregulated genes, and inverted triangles indicate significantly downregulated genes. (C–F) Contents of jasmonates in flower buds with stamen primordia (SP) and in anthers at meiocyte (ME) and microspore (MS) stages. OPDA content (C), JA content (D), JA-ILE content (E), MEJA content (F). In (C–F), data are shown as mean ± standard deviation. Three biological repeats were performed. ^*^P < .05, ^**^P < .01, ^***^P < .001, ^****^P < 0.0001 (Student’s *t*-test).

JA metabolic process and response to JA were enriched among DEGs in g1-cluster 1 and cluster 2, which showed a higher expression level in group 1 (SP1, SP2) organs of G1 + HBP compared with HBP (Fig. 2, Supplementary Data Fig. S6). JA pathway-related genes were also enriched in the pink module, which was highly correlated with SP2 of G1 + HBP (Supplementary Data Set S7). We profiled all the genes involved in JA biosynthesis and signal transduction. The majority of JA biosynthesis-related genes, including lipoxygenase (LOX) (12/15), allene oxide synthase (AOS) and cyclase (AOC) (5/5), and 12-OPDA reductase (OPR) (5/6), were all upregulated in group 1 (SP1, SP2) organs of G1 + HBP compared with HBP, as well as JA signal transduction-related genes, including *JASMONIC ACID CARBOXYL METHYLTRANSFERASE* (*CgJMT*), *CgMYC2* and *JASMONATE-ZIM-DOMAIN PROTEIN* (*CgJAZs*) (7/8) (Fig. 4A and B). qRT–PCR showed that expression of JA biosynthesis and signal transduction-related DEGs was also upregulated in SPF, MEA, and MSA of G1 + HBP compared with HBP (Supplementary Data Fig. S9). Correspondingly, the contents of *cis*(+)-12-oxophytodienoic acid (OPDA), JA, and jasmonoyl-l-isoleucine (JA-ILE) were upregulated in SPF, MEA, and MSA in G1 + HBP compared with HBP (Fig. 4C–F). In addition, *CgMYB21* that respond to JA signals and function in stamen development [41] was downregulated in MS of G1 + HBP compared with HBP (Fig. 4B).

Auxin biosynthesis process was enriched among DEGs in g1-cluster 2, which showed downregulated expression level in group 1 (SP1, SP2) organ of G1 + HBP compared with HBP (Fig. 2). IAA biosynthesis-related genes *CgTAR2* and *CgYUC4* were both downregulated in SP2 of G1 + HBP compared with HBP, as well as IAA signal transduction-related genes *IAA CARBOXYLMETHYLTRANSFERASE 1* (*CgIAMT1*) and *METHYL ESTERASE 1* (*CgMES1*) (Supplementary Data Fig. S10A and B). In addition, *ARF1*/*3*/*9*/*16* were downregulated in group 3 (MS) tissue of G1 + HBP compared with HBP (Supplementary Data Fig. S10B). Among the auxin biosynthesis and signal transduction-related DEGs, *CgTAR2*, *CgYUC4*, *CgIAMT1*, and *CgARF* were downregulated in SPF, MEA, and MSA of G1 + HBP compared with HBP (Supplementary Data Fig. S9). Correspondingly, the contents of IAA, l-tryptophan, tryptamine, OxIAA, and MeIAA were downregulated in SPF, MEA, and MSA of G1 + HBP compared with HBP (Supplementary Data Fig. S10C–G). The expression of the majority of stamen development-related genes was affected by exogenous application of JA and yucasin (a protein inhibitor of YUCCA) in flower buds of HBP. Among them, *CgAP3.2* was upregulated, while *CgPI.1*, *CgPI.2*, *CgSEP3*, *CgTGA9*, and *CgTGA10* were downregulated, which coincided with the expression pattern changes of these genes in G1 + HBP compared with HBP (Supplementary Data Fig. S11). In summary, overactive JA biosynthesis and signal transduction, together with inactive auxin biosynthesis and signal transduction, might have caused stamen development defects in the male-sterile G1 + HBP.

### Disrupted mitochondrial function in stamen organ and cell types of G1 + HBP

The mitochondrion is the primary organelle for the respiratory chain, OXPHOS, and TCA reactions, which provide energy and metabolites for organism activity and development. We previously demonstrated that the mitochondrial genome of G1 + HBP was from its CMS callus parent Satsuma mandarin G1, indicating that male sterility in G1 + HBP is a result of nuclear–mitochondrial incompatibility [30–32]. Significantly, energy metabolism-related processes, mitochondria, and respirasome components were enriched among DEGs in g2- and 3-cluster 2 and g2- and 3-cluster 3 with lower expression level in group 2 (PMC, TE) cells of G1 + HBP compared with HBP (Fig. 2).

**Figure 5:**
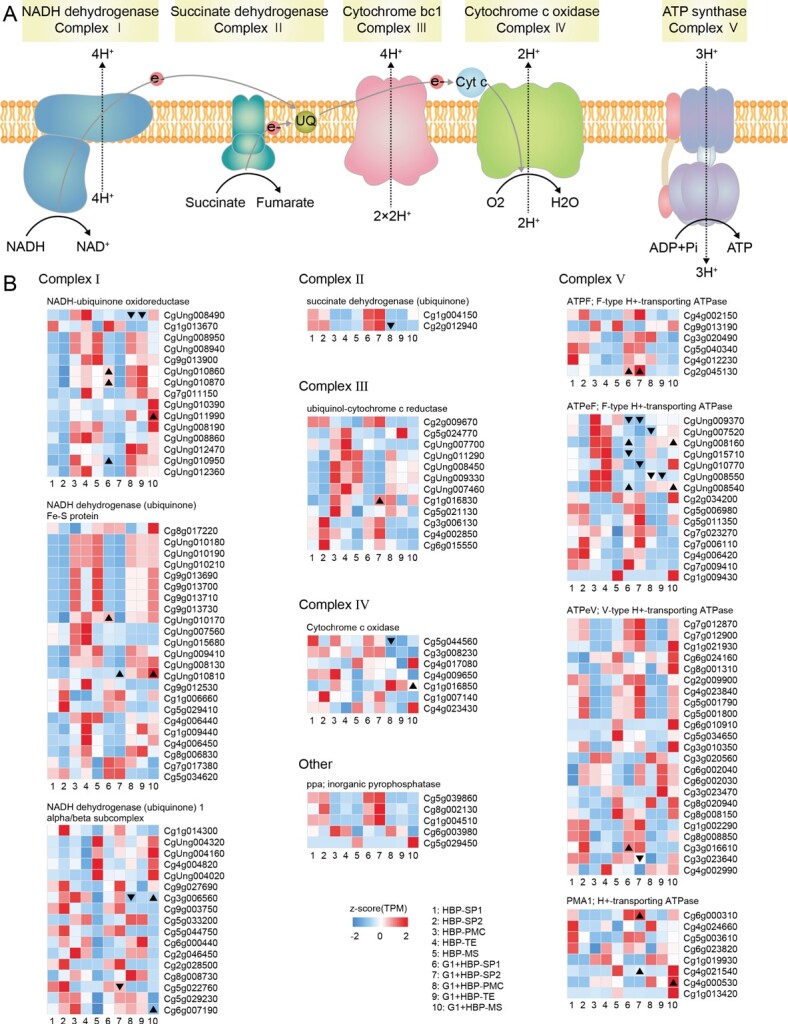
Expression level of oxidative phosphorylation genes in stamen organ and cell types of HBP and G1 + HBP. (A) Illustration of oxidative phosphorylation. (B) Expression level of oxidative phosphorylation genes in stamen organ and cell types of HBP and G1 + HBP. Color in the heat map indicates normalized TPM using *z*-scores. DEGs are marked with triangles; regular triangles indicate significantly upregulated genes and inverted triangles indicate significantly downregulated genes.

Genes comprising the five mitochondrial respiratory chain complexes and required for OXPHOS had specific expression patterns in stamen organ and cell types in fertile HBP (Fig. 5). Among them, a few genes (27/133) were differentially expressed between HBP and G1 + HBP, and there were more upregulated genes in SP1 (eight), SP2 (five), and MS (eight) of G1 + HBP than those downregulated (two, four, and none), and more downregulated genes in PMC (six) and TE (two) of G1 + HBP than upregulated genes (none and none) (Fig. 5). These DEGs were distributed in all OXPHOS complexes (Fig. 5), indicating that OXPHOS might have been affected in the sterile cybrid G1 + HBP. A majority of the TCA cycle-related genes were expressed at a higher level in stamen primordia (12/34) and meiocytes (18/34) compared with microspores (4/34) in both HBP and G1 + HBP (Supplementary Data Fig. S12). Although only 11 TCA-cycle related genes were differentially expressed between HBP and G1 + HBP, they were distributed in most of the steps (six of eight) of the TCA cycle, indicating that the TCA cycle might have been disturbed in the sterile cybrid G1 + HBP. There were more upregulated genes in SP2 (seven) of G1 + HBP than downregulated genes (one), while only *SUCCINATE DEHYDROGENASE 2-2* (*CgSDH2-2*) and *ISOCITRATE DEHYDROGENASE 1* (*CgIDH1*) were dramatically downregulated in PMC of G1 + HBP (Supplementary Data Fig. S12). In summary, we suggest that the mis-regulated mitochondria-related genes, especially those of OXPHOS and TCA, might have disturbed mitochondrial functions in stamen organ and cell types of G1 + HBP and be responsible for male sterility.

## Discussion

### Disrupted jasmonic acid and indoleacetic acid homeostasis might have resulted in the stamen initiation defect in G1 + HBP

The stamen primordium is the initial tissue of the stamen that can differentiate to filament and anther [42]. The initiation of stamen primordia was deficient in G1 + HBP (Fig. 1, Supplementary Data Fig. S1), and a majority of stamen development-related genes were downregulated in stamen primordia of G1 + HBP compared with HBP, especially ABCE model genes, which determine flower organs [43], like the upregulated *CgAG* and *CgSEP1.1* and downregulated *CgAP2.2* and *CgSEP3* in SP1 of G1 + HBP (Figs 2 and 3). Defective petals and stamens in G1 + HBP are similar to the typical phenotypes controlled by B-class genes in model plants [44, 45]. It was reported that *CitAP3.3* (*CitMADS8*) failed to complement the *ap3* mutant in *Arabidopsis* [46]. In our results, *CgAP3.2* was the only differentially expressed gene among the three B-class *CgAP3* members, and it was a newly identified *AP3* homologous of an independent *Citrus* genus branch (Fig. 3, Supplementary Data Fig. S8). Together with the protein interaction models between CgAP3 and ABCE model genes that are different from those in *Arabidopsis* (Fig. 3D), we suggested that *CgAP3.2* might be important for the defect of stamen development in G1 + HBP.

Changes in JA and IAA contents, together with the altered expression pattern of JA and IAA biosynthesis and signaling-related gene in SP1 and SP2, indicated the disruption of hormone homeostasis derived from *de novo* biosynthesis (Fig. 4, Supplementary Data Fig. S10). In plants, JA and IAA biosynthesis and signal transduction process, together with their crosstalk, were required for stamen development [47–49]. Application of MeJA solution could result in the expression change of ABCE model genes in oilseed [50]. Auxin signaling could inhibit the expression of PI-like gene *PeMADS6* in orchid [51]. Transcription factors, including MYBs and ARFs, usually respond to JA and IAA signaling, respectively, and possibly function in stamen development [52–54]. The majority of *MYB* genes were upregulated in flower buds and anthers of G1 + HBP compared with HBP, while *ARF* genes were downregulated, which coincided with the hormone content change patterns in G1 + HBP (Supplementary Data Fig. S10B). In addition, *CgMYB21* was downregulated in MS of G1 + HBP compared with HBP, and *CgARF3*/*4* were upregulated in SP1 and SP2 of G1 + HBP (Fig. 4B). The disrupted JA and IAA metabolic process and the changed expression of stamen primordia development-related genes might be responsible for the stamen initiation defects in G1 + HBP.

### The disordered mitochondria related processes might have caused deficiency of meiocytes and microspores in G1 + HBP

Meiocytes in anthers undergo meiosis to generate haploid microspores, followed by mitosis to form mature pollen [55, 56]. We previously observed meiosis I, tapetum and pollen wall defects in G1 + HBP [34]. Male meiosis might be dependent on tapetum functions [57], as transcripts of tapetum-related genes *DYT1* and *TDF1* were detectable in both tapetum and meiocytes of *Arabidopsis* [58, 59]. In our results, meiosis-related genes showed higher expression in meiocytes of G1 + HBP. Tapetum and pollen development-related genes were upregulated in meiocytes of G1 + HBP as well. However, pollen wall-related genes were downregulated in MS of G1 + HBP (Supplementary Data Set 6). Thus, the altered expression of essential genes involved in meiosis and microspore development might have caused the deficiency of meiocytes and microspores in G1 + HBP.

Energy metabolic and respiration process in mitochondria were enriched among DEGs in meiocytes of G1 + HBP (Fig. 2). The mitochondria have been elucidated as the energy and metabolite providers for meiocytes and microspore development [60], and mitochondrial shape, size, and location were changed during meiosis [61]. In addition, transport and mitochondrial DNA replication were enriched among DEGs in microspores of G1 + HBP (Fig. 2). In rice and maize, transport and nucleotide metabolism-related genes were preferentially expressed in microspores [62, 63]. In summary, the mitochondria-related processes were obviously disordered in meiocytes and microspores, which indicated the direct effects of mitochondria on meiosis and microspore development of G1 + HBP, which might have caused deficiency of meiocytes and microspores in G1 + HBP.

### Conceivable model of nucleus–mitochondria in the regulation of stamen development in G1 + HBP

The mitochondrial genome of G1 + HBP was derived from its CMS parents [31, 32]. We previously demonstrated that the content of TCA cycle intermediates was disturbed in G1 + HBP [34]. In this study, the altered expression of OXPHOS and TCA-related genes indicated the dysfunction of mitochondria from stamen primordia initiation to microspore development in G1 + HBP (Fig. 5, Supplementary Data Fig. S12). Together with the distinct expression pattern of nuclear DEGs from stamen primordia initiation to microspore development (Fig. 2, Supplementary Data Fig. S6), the results suggest that the male sterility of G1 + HBP might be attributed to the abnormal spatiotemporal interplay between mitochondria and nucleus, which resulted in stamen primordium, meiocyte, and microspore development defects.

Interestingly, hormone and mitochondrial signaling seem to interact in stamen primordia of G1 + HBP. The dysfunction of mitochondria may change IAA levels and disrupt IAA signal transduction to regulate the expression of nuclear genes, while the effects of mitochondria on JA are less known [11, 12, 64]. JA biosynthesis initiated in chloroplast plastids, and the photosynthetic products in the chloroplast could serve as substrates for biosynthesis of JA [65, 66]. The intimate linkage of respiration in mitochondria and photosynthesis in chloroplasts has long been recognized, and inhibition of mitochondrial function could alter photosynthetic gene expression [67–69]. In our results, JA, TCA, and photosynthesis-related processes were all enriched in stamen primordia (Supplementary Data Fig. S7). DEGs related to these processes shared a similar expression pattern in g1-cluster 1 (Fig. 2), which indicated that the disturbed JA metabolism in stamen primordia of G1 + HBP was associated with abnormal photosynthesis and mitochondrial functions. Therefore, we suppose that mitochondria might affect JA metabolic process indirectly through photosynthesis in chloroplasts, and thus inhibit stamen primordium development in G1 + HBP.

**Figure 6:**
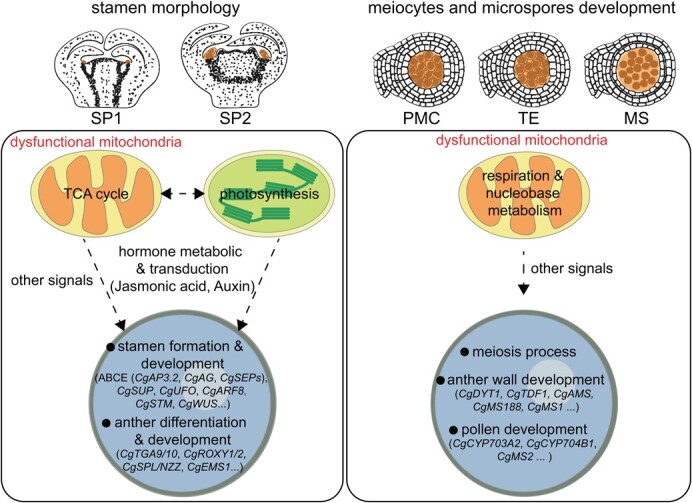
Conceivable model of nucleus–mitochondria interaction in the regulation of stamens in G1 + HBP. During stamen primordium development, dysfunctional mitochondria might affect photosynthesis in chloroplasts and thus cause disorder in JA biosynthesis, and together with IAA and other mitochondria retrograde signals affect the expression of nuclear genes involved in stamen development. During meiosis, dysfunctional mitochondria might affect expression levels of nuclear genes involved in anther development through disrupted respiration. During microspore development, dysfunctional mitochondria might affect microspores through nucleobase metabolism and transduction. Taking the results together, the disrupted nucleus–mitochondria interactions might have caused abnormal stamen development and male sterility in G1 + HBP.

In summary, our results highlight the dysfunction of mitochondria and nucleus in stamen organ and cell types of G1 + HBP at transcriptome levels. In stamen primordia, dysfunctional mitochondria might disrupt IAA metabolism directly, and affect JA metabolism indirectly through the abnormal photosynthesis in chloroplasts, which together changed the expression of stamen development-related genes (such as ABCE model genes) and interfered in stamen and flower morphology (Fig. 6). In meiocytes, the disrupted energy metabolism-related process of dysfunctional mitochondria might have caused expression changes of meiosis and tapetum-related genes to disrupt the meiosis process (Fig. 6). In microspores, dysfunctional mitochondria might disrupt nucleobase metabolism and transport, resulting in expression changes of pollen wall development-related genes, and thus interfering with pollen wall formation (Fig. 6). Our results provide insight into the mechanisms of stamen development in citrus, and propose nucleus–mitochondria regulation of deficient stamen development in the male-sterile cybrid pummelo G1 + HBP, which would be exploited in citrus seedless breeding.

## Materials and methods

### Plant materials and sample preparation

Trees of male-sterile cybrid pummelo G1 + HBP and the fertile mesophyll parent HBP were grown in the field of the National Citrus Breeding Center at Huazhong Agricultural University. Flower buds were collected from three trees as individual biological replicates. Flower buds with diameter ≤5 mm were collected entirely, while anthers were dissected from flower buds with diameter >5 mm; they were snap-frozen in liquid nitrogen and stored at −80°C. The fresh samples were washed with phosphate-buffered saline and fixed in a mixture of ethanol and acetic acid (3:1), followed by dehydration with an ethanol gradient. The dehydrated samples were placed in a sucrose gradient for cryoprotection (10 and 15%) before being embedded in optimal cutting temperature (O.C.T.) compound (Sakura Tissue-Tek, USA) and snap-frozen in liquid nitrogen for cryosection immediately or storage at −80°C.

### Laser capture and RNA sequencing of stamen organ and cell types

The embedded samples were sectioned at 10 μm thickness using a cryostat (Leica CM1950, Germany), and mounted on PEN membrane glass slides (Leica LCM0522, Germany). The frozen sections were immediately dehydrated successively with an ethanol and xylene gradient before being air-dried. Stamen organ and cell types were captured using a laser microdissection system (Leica LMD7000, Germany), with three biological replicates each containing sections from >10 flower buds.

Total RNA was extracted and purified from laser microdissection (LMD) samples using an Arcturus PicoPure™ RNA Isolation Kit (Applied Biosystems™, USA) and an RNase-Free DNase Set (Qiagen, Germany). RNA quality was examined using a 2100 Bioanalyzer (Agilent Technologies, Germany) with an RNA 6000 Pico Kit (Agilent Technologies, Germany). RNA was amplified using the Smart-Seq2 method. An oligo-dT primer was introduced into the reverse transcription reaction for first-strand cDNA synthesis, followed by PCR amplification to enrich the cDNA and purification using Magbeads. The cDNA was then used for library preparation, followed by PE150 sequencing on the Illumina platform (Annoroad, Beijing, China).

### RNA sequencing data analysis

The RNA-seq raw reads were quality-controlled by FastQC (https://www.bioinformatics.babraham.ac.uk/projects/fastqc/), followed by adapter and low-quality sequence removal using Trimmomatic. The clean reads were aligned to the pummelo genome (*Citrus grandis* v1.0, http://citrus.hzau.edu.cn/) using HISAT2, and the aligned reads were assembled into transcripts using StringTie [70]. Read counts for each unigene were normalized to transcripts per million (TPM). The expressed unigenes were defined as those with TPM >1.0 in at least two of three biological replicates. The raw data can be found in the NCBI Sequence Reads Archive (SRA), BioProject ID: PRJNA938902.

Pearson coefficients were calculated and principal component analysis (PCA) was performed using the R program (https://www.r-project.org/) based on TPM. Differentially expressed genes (DEGs) were identified using an exact test provided in the DESeq2 package [71], with *P* value ≤.05. DEGs were clustered according to expression patterns using soft clustering with the Mfuzz package [72]. Membership >0.5 was defined as significantly affiliated with one cluster. Co-expression network modules were identified using the WGCNA (weighted gene co-expression network analysis) package [73]. The unigenes were screened according to coefficient of variation (CV) >0.5 among all tissues, and the average expression of selected annotated unigenes was used for WGCNA analysis. The co-expression modules were obtained by an automatic network construction function (blockwiseModule) with modified parameters (power 12, TOMType unsigned, minModuleSize 50, mergeCutHeight 0.1). Unigenes with the highest degree of connectivity within a module were assigned as intramodular hubgenes. The global gene co-expression network was visualized using Cytoscape 3.9 [74].

### Gene annotation and functional enrichment analysis

The unigenes were mapped to the pummelo genome using StringTie, and then used for a BLASTP search (E value <1e^−5^) against proteins of *Arabidopsis* (TAIR, https://www.arabidopsis.org/) and UniProt (https://www.uniprot.org/) for gene function and gene ontology (GO) annotations. The eggNOG-mapper [75] was also used for GO annotation. GO enrichment and gene set enrichment analysis (GSEA) were performed using the clusterProfiler package [76], by applying a hypergeometric test with false discovery rate (FDR) <0.05 to identify significantly enriched items.

### Identification and phylogenetic analysis of ABCE model homologous genes

The citrus protein sequences (*Citrus clementina* v1.0, *C. grandis* v1.0, *C. sinensis* v3.0, and *Poncirus trifoliata* v1.0) were downloaded from the Citrus Pan-genome to Breeding Database (CPBD, http://citrus.hzau.edu.cn/). The protein sequences of *Solanum lycopersicum* (ITAG4.0) and *Fragaria vesca* (v4.0.a2) were downloaded from Phytozome v12 (https://phytozome-next.jgi.doe.gov/). The protein sequences of *Oryza sativa* were downloaded from RAP-DB (https://rapdb.dna.affrc.go.jp/). The orthologs of ABCE model genes were retrieved from a BALSTP search (E value <1e^−5^) against *Arabidopsis* protein sequences in TAIR. Protein sequences of AP3 were downloaded from NCBI (https://www.ncbi.nlm.nih.gov/) and UniProt. Full-length protein sequences of genes were aligned using the Clustal Omega program with default settings, and the phylogenetic tree was constructed using IQ-TREE with the maximum likelihood method and bootstrap analysis (1000 replicates).

### Sequence analysis and yeast two-hybrid assay

Total RNA was extracted from flower organs and tissues using TRIzol reagent (Sigma, Germany) and then reverse-transcribed using the HiScript II 1st Strand cDNA Synthesis Kit (Vazyme, China). The yeast two-hybrid (Y2H) assay was performed as previously reported [77]. The coding sequence of *CgAP3* was inserted into pGBKT7 as the bait vector, and ABCE homologous genes were individually inserted into pGADT7 as the prey vector. The pair of bait vector and prey vector were co-transformed into the Y2HGold yeast strain (Clontech, USA) to examine self-activation and interaction. The Y2H assay primers are listed in Supplementary Data Table S1.

### Determination of indole-3-acetic acid and jasmonic acid contents

Flower buds at stamen primordia development stage (SPF), anthers at meiosis stage (MEA) and anthers at microspore development stage (MSA) were used for indole-3-acetic acid (IAA) and jasmonic acid (JA) measurement. Contents of the phytohormones IAA and JA were measured by the AB Sciex QTRAP 6500 LC–MS/MS platform (MetWare, Wuhan, China). The experiment was performed with three biological replicates, with flower buds from one tree as an individual replicate. The flower buds and anthers were ground into powder and then were separately dissolved in 1 ml methanol/water/formic acid (15:4:1, v/v/v) followed by addition of 10 μl internal standard mixed solution (100 ng/ml). The supernatant was collected after vortexing and centrifugation, and then evaporated to dryness and dissolved in 100 μl 80% methanol (v/v), and finally filtered using a 0.22-μm membrane filter. The extracting solution was measured using an LC–ESI–MS/MS system (UPLC, SCIEX ExionLC™ AD, USA; MS, Applied Biosystems 6500 Triple Quadrupole, USA). Hormone standards for jasmonoyl-l-isoleucine (JA-ILE), *cis*(+)-12-oxophytodienoic acid (OPDA), 2-oxindole-3-acetic acid (OxIAA), IAA, methyl indole-3-acetate (MeIAA) were purchased from OlChemlm (Czech Republic); hormone standards for JA, methyl jasmonate (MeJA), tryptamine, and l-tryptophan (TRP) were purchased from RHAWN (China).

### Quantitative reverse transcription–PCR

Total RNA was reverse-transcribed for qRT–PCR using the HiScript II Q RT SuperMix for qPCR kit (Vazyme, China). *CgACTIN* was used as the endogenous reference gene. qRT–PCR was performed as described previously using a LightCycler 480 (Roche, Switzerland) [77]. Three independent biological replicates were conducted. The primers used for qRT–PCR are listed in Supplementary Data Table S1.

### Statistical analyses

Statistical analyses were performed using R. Pairwise comparisons were conducted using Student’s *t*-test.

## Acknowledgements

This research was financially supported by the Ministry of Science and Technology of China (2022YFF1003101), the National Natural Science Foundation of China (31530065, 31820103011, 32202451), and the Foundation of Hubei Hongshan Laboratory (2021hszd009).

## Author contributions

X.M.W. and W.W.G. conceived and supervised the research; N.J. performed the experiments and analyzed the data with contributions from M.Q.F., L.C.C., L.H.K., C.C.L., Z.P.Y., R.W., and K.D.X; N.J. and X.M.W. wrote the manuscript with contributions from all co-authors.

## Data availability

The raw data that support the findings of this study are openly available in NCBI database under the BioProject accession number PRJNA938902.

## Conflict of interest

The authors declared that they have no conflicts of interest.

## Supplementary data

Supplementary data is available at *Horticulture Research* online.

## Supplementary Material

Web_Material_uhad105
