# Supplementary material for: Spatiotemporal profiles of gene activity in stamen delineate nucleo-cytoplasmic interaction in a male-sterile somatic cybrid citrus
Source: Hortic Res. 2023 May 12;10(7):uhad105. doi: 10.1093/hr/uhad105 (PMC10419853; doi:10.1093/hr/uhad105)
Supplement: Web_Material_uhad105 [file web_material_uhad105.zip › Supplemental Figure S1-S12.docx]

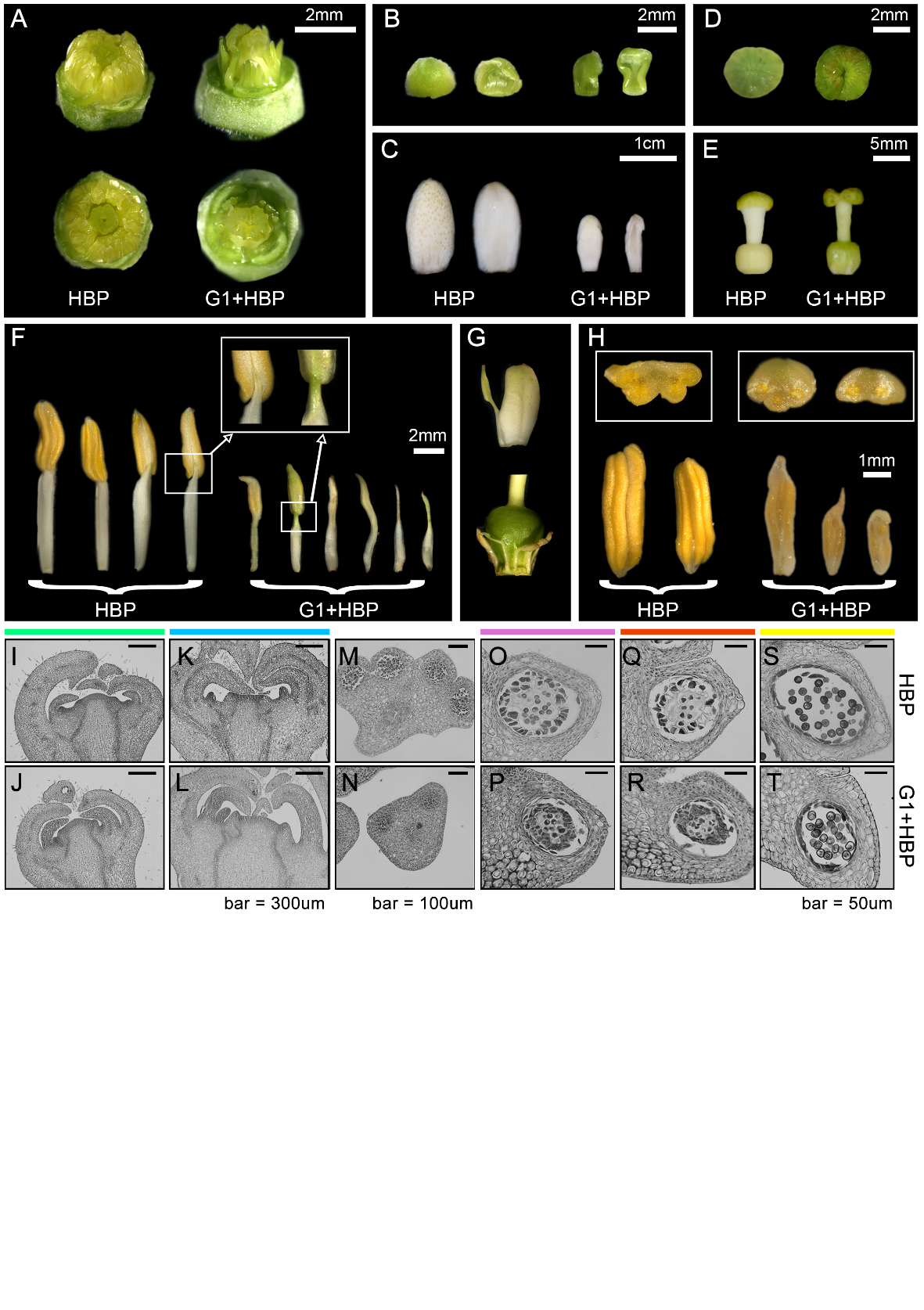


**Supplemental** **Figure S1.** **Morphological and histological observation of flower organs in HBP and G1+HBP.** A. Enlarged stamen primordia on receptacle. B. Petals during stamen primordia development. C. Petals at pollen maturation. D. Stigma at pollen maturation. E. Carpel during flower bloom. F. Stamen at pollen maturation. G. Stamen fused with petal or carpel in G1+HBP. H. Anthers and transection of anthers. I-T. Histological observation of flower bud and anther. I,J SP1; K,L SP2; M,N anther; O, P PMC; Q, R TE; S, T MS. Color lines on the top of I-T represents stamen organ and cell types; springgreen, skyblue, orchid, orangered and yellow represent SP1, SP2, PMC, TE and MS, respectively.


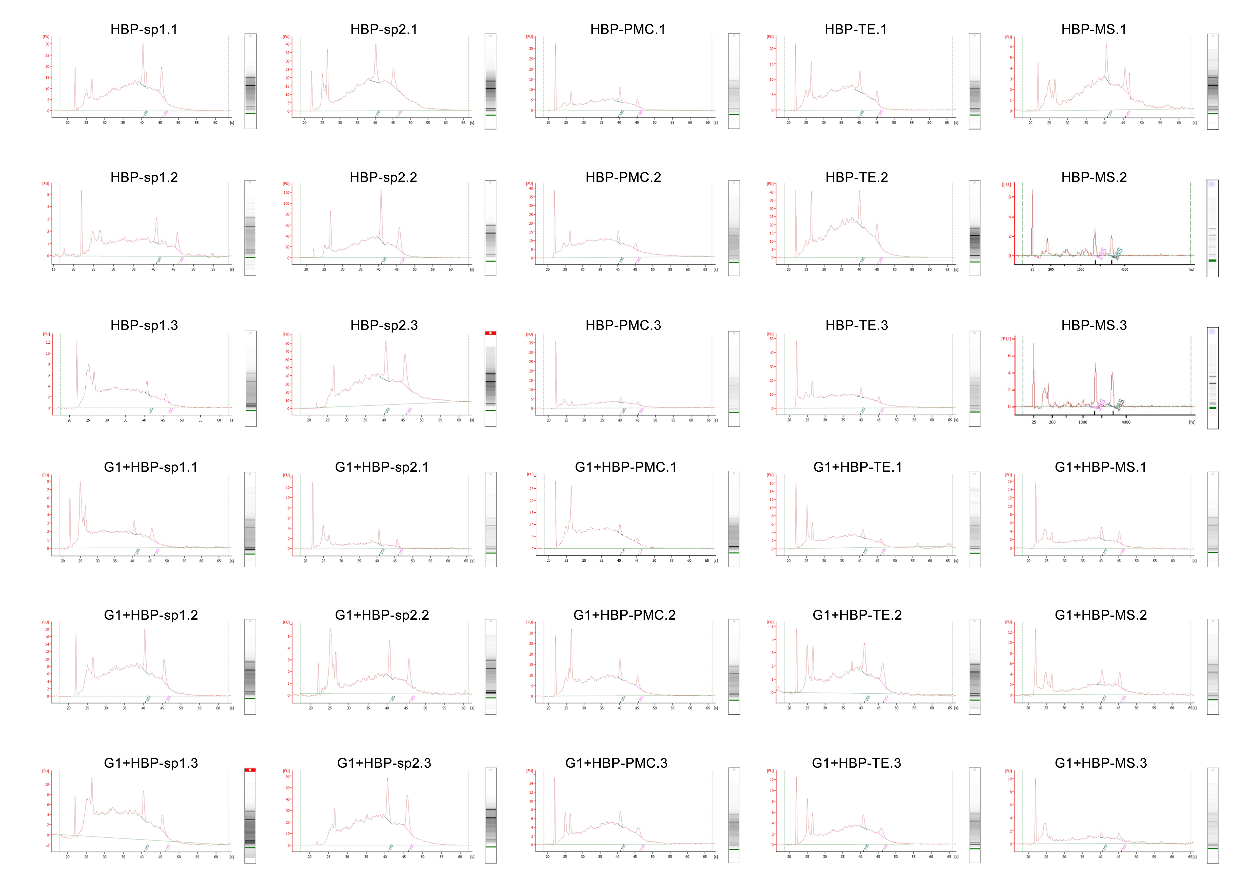


**Supplemental** **Figure S2.** RNA quality of stamen organ and cell types of HBP and G1+HBP that captured using laser microdissection.


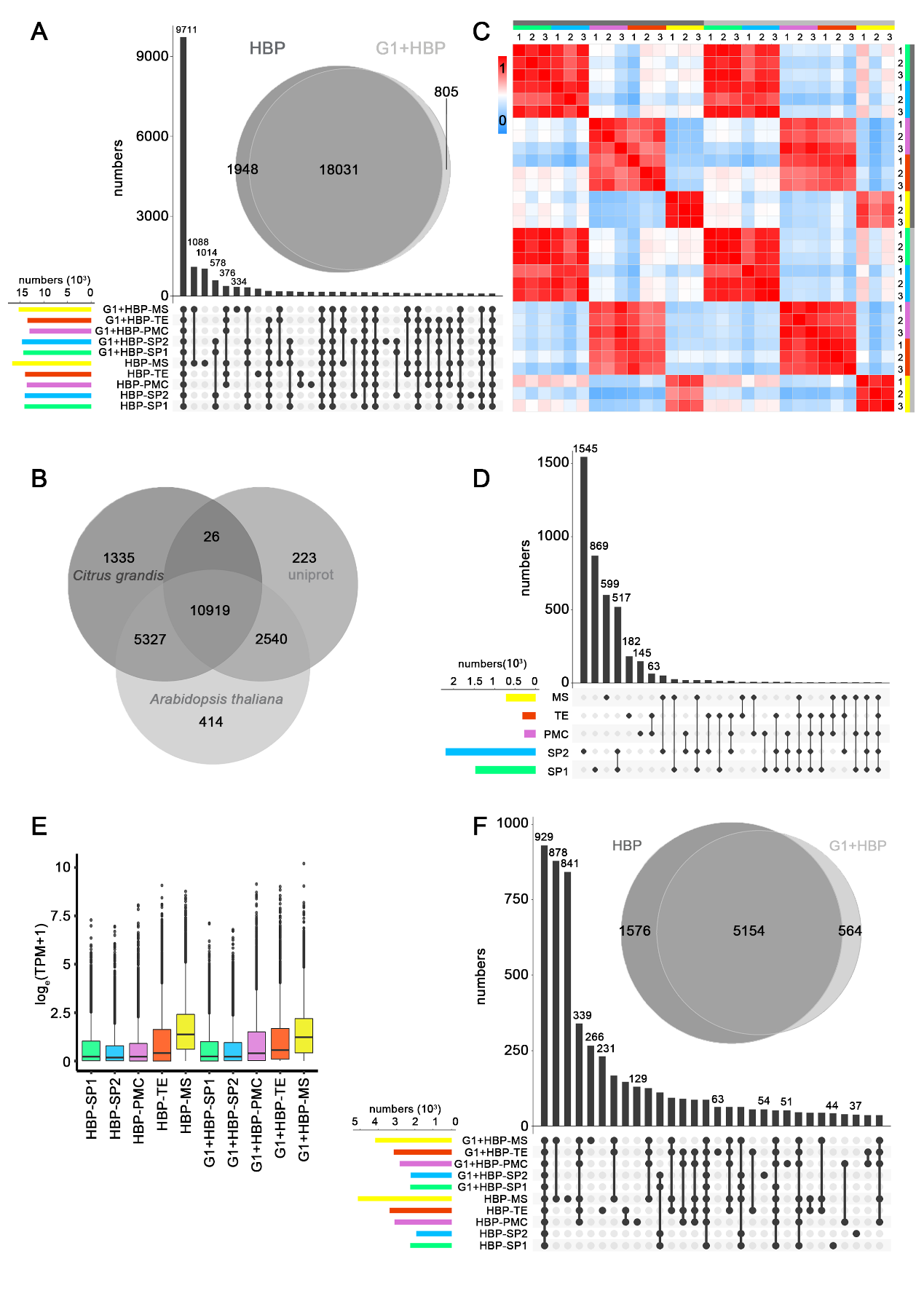


**Supplemental** **Figure S3. Statistic of gene expression in stamen organ and cell types of HBP and G1+HBP.** A. Upset plot shows the intersection of unigenes in stamen organ and cell types of HBP and G1+HBP, subset numbers of the top six histogram were marked on the top. Venn diagram shows the intersection of total unigenes of stamen organ and cell types in HBP and G1+HBP. B. Annotation statistic of expressed unigenes in stamen organ and cell types derived from LMD. C. Pearson correlationof unigenes in stamen organ and cell types of HBP and G1+HBP. Color lines in C represent stamen organ and cell types corresponding to the color represented in A and F, springgreen represents SP1, skyblue represents SP2, orchid represents PMC, orangered represents TE, yellow represents MS, darkgrey represents HBP, lightgrey represents G1+HBP. D. Upset plot shows the intersection of DEGs in stamen organ and cell types of HBP and G1+HBP, subset numbers of the top seven histogram were marked on the top. E. Expression abundance of unigenes preferentially expressed in stamen organ and cell types, box horizontal line represents median, box limits represent upper and lower quartiles, y axis indicates expression value (score as log_e_ (TPM+1)). F. Upset plot shows the intersection of unigenes preferentially expressed in stamen organ and cell types of HBP and G1+HBP, subset numbers of top six histogram and sample unique histogram were marked on the top. Venn diagram shows the intersection of total unigenes of stamen organ and cell types in HBP and G1+HBP.


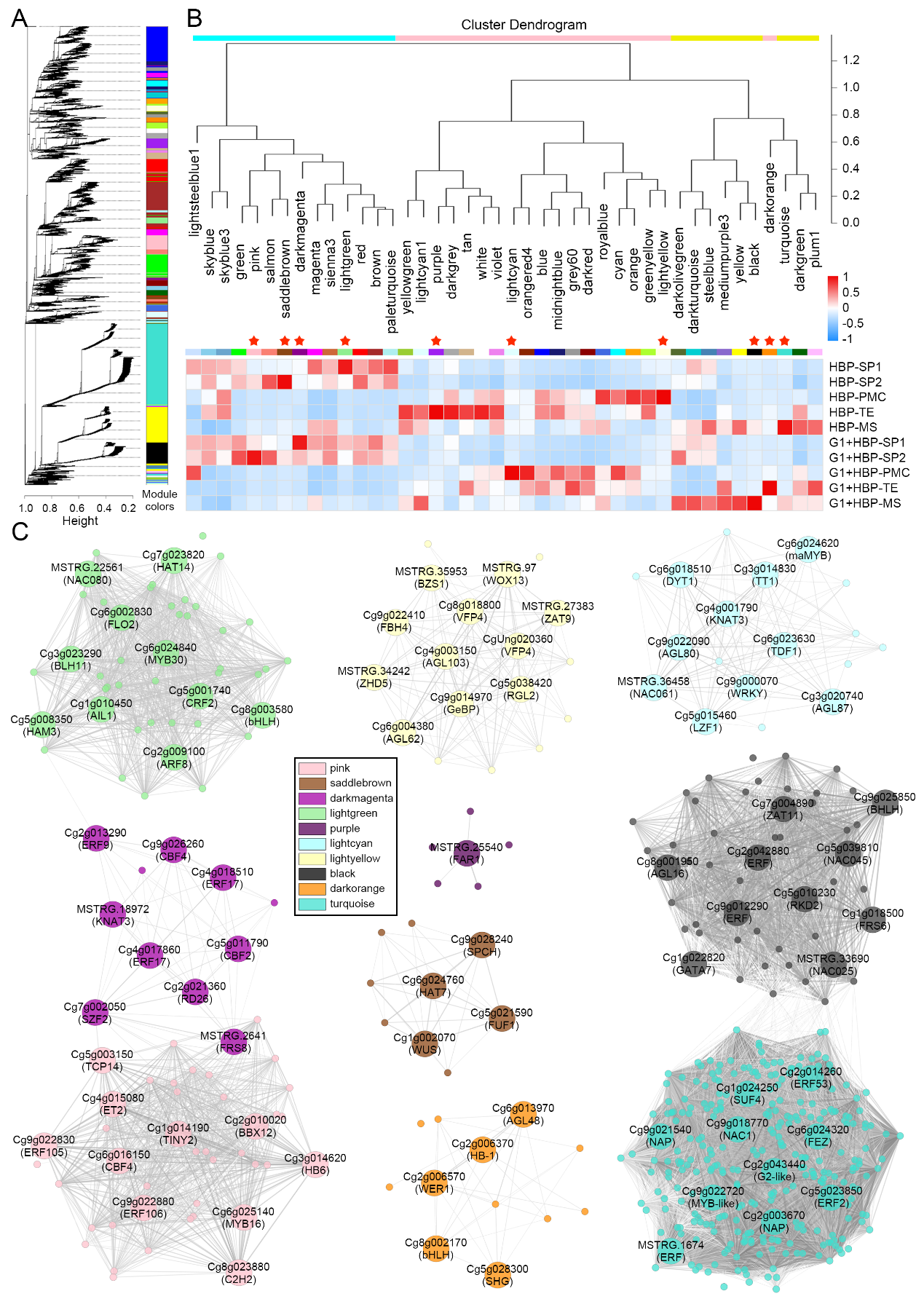


**Supplemental** **Figure S4. Weighted gene co-expression network analysis (WGCNA) for stamen organ and cell types of HBP and G1+HBP.** A. Hierarchical cluster dendrogram showing co-expressed modules identified by WGCNA. Each leaf on the tree represents one gene, the major tree branches constitute 42 merged modules (based on a threshold of 0.1), marked with different colors. B. Hierarchical cluster dendrogram of modules and association between modules and tissues. Each leaf on the tree represents one module, the major tree branches were grouped into three classes, marked with color lines on the top. Cyan represents group1 (SP1 and SP2) organ, pink represents group2 (PMC and TE) cells, yellow represents group3 (MS) cells. The bottom heatmap indicates the association between modules and tissues, color indicates the Pearson correlation coefficient, the top ten associated modules are marked with star on top of the heatmap. C. Construction of the co-expression network of transcription factors in ten organ and cell types associated modules: lightgreen (HBP-SP1), saddlebrown (HBP-SP2), lightyellow (HBP-PMC), purple (HBP-TE), turquoise (HBP-MS), darkmagenta (G1+HBP-SP1), pink (G1+HBP-SP2), lightcyan (G1+HBP-PMC), darkorange (G1+HBP-TE), black (G1+HBP-MS). The large circles represent hub transcription factors with high connectivity.


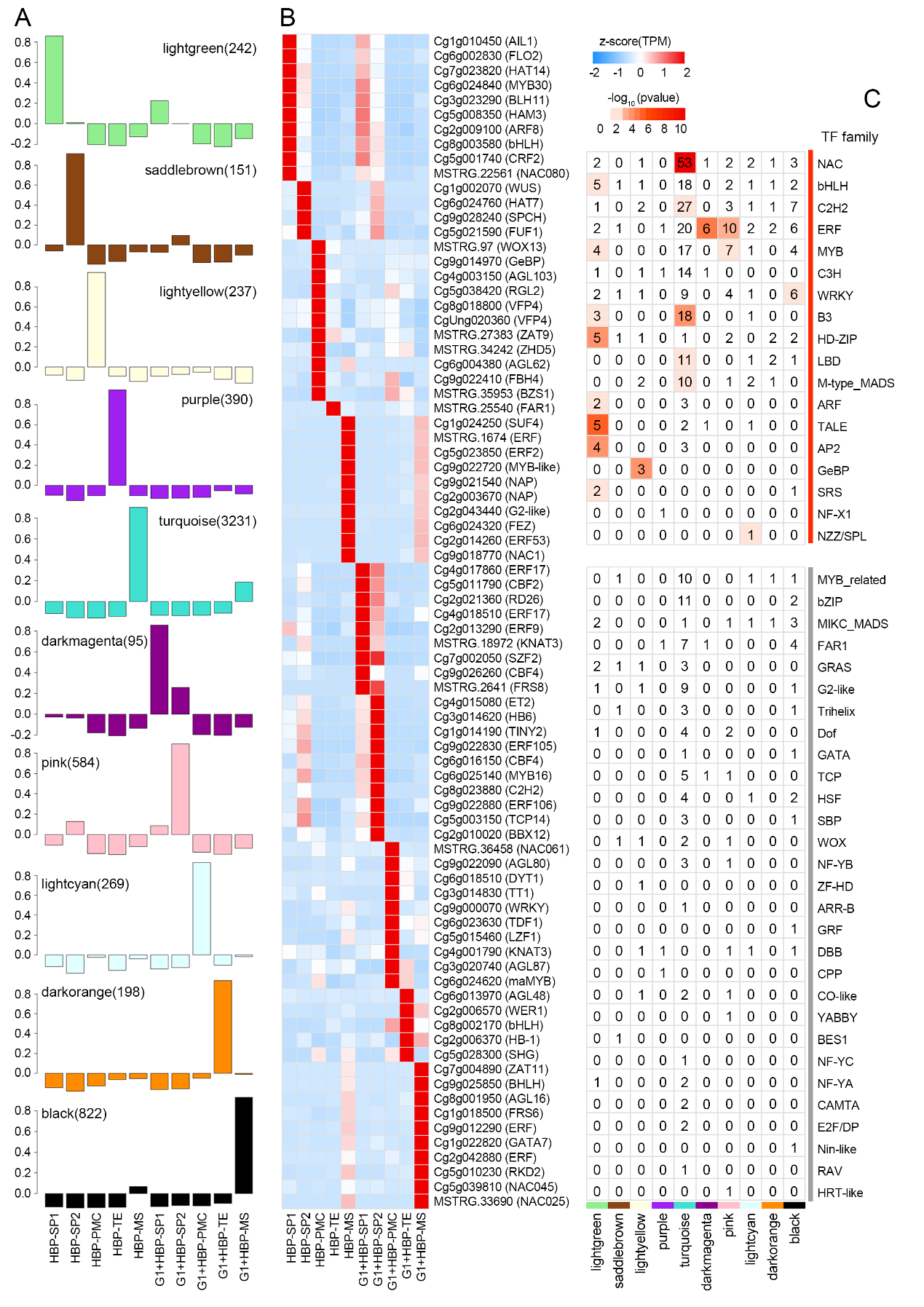


**Supplemental** **Figure S5. Expression level of unigenes in stamen organ and cell types associated modules identified by WGCNA.** A. Eigengene expression profiles in stamen organ and cell types associated modules (lightgreen (HBP-SP1), saddlebrown (HBP-SP2), lightyellow (HBP-PMC), purple (HBP-TE), turquoise (HBP-MS), darkmagenta (G1+HBP-SP1), pink (G1+HBP-SP2), lightcyan (G1+HBP-PMC), darkorange (G1+HBP-TE), black (G1+HBP-MS)). B. Expression level of hub transcription factors with high connectivity, color in heatmap indicates normalized TPM using z-score. C. Enrichment analysis of transcription factor families in stamen organ and cell types associated modules, x-axis indicates stamen organ and cell types associated modules, y-axis indicates transcription factor families, color indicates significance level (score as -log_10_ (pvalue)) using hypergeometric analysis with R, the number of transcription factor family members in modules is exhibited in the box.


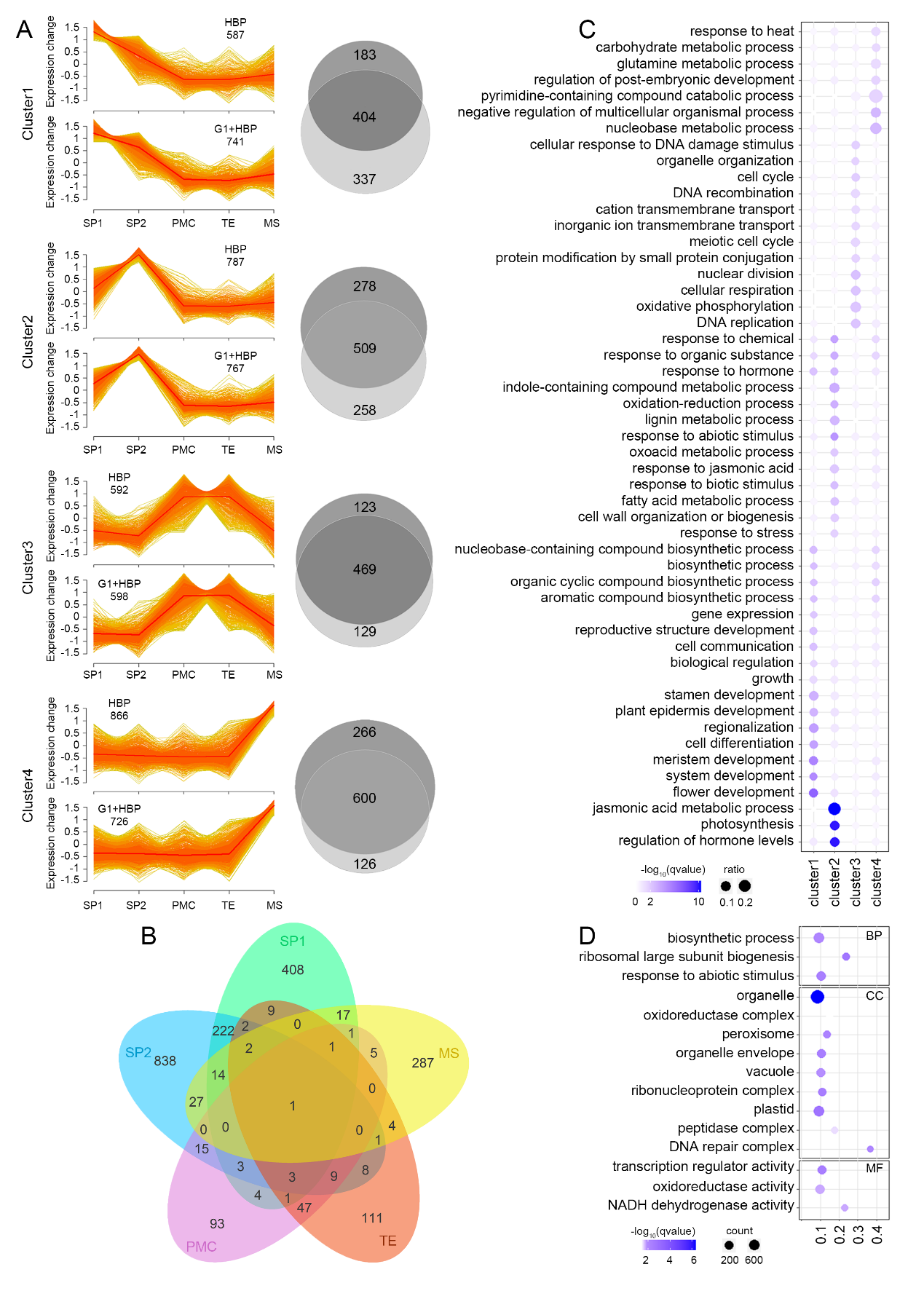


**Supplemental Figure S6. Expression pattern and gene ontology (GO) enrichment analysis of DEGs in stamen organ and cell types of HBP and G1+HBP.** A. Clustering analysis of DEGs in stamen organ and cell types of HBP and G1+HBP. Normalized TPM is used for c-means soft clustering with Mfuzz. DEGs in both HBP and G1+HBP were grouped into four clusters, Venn diagrams indicate the intersection of DEGs with the same expression pattern in HBP and G1+HBP, unigenes with membership > 0.5 are considered significantly affiliated to the cluster, the y axis indicates the normalized transcripts per million (TPM) using z-score. B. GO enrichment analysis of DEGs with similar expression pattern, with biological process exhibited; x-axis indicates clusters, y-axis indicates enriched GO items, size indicates percentage of identified genes in background (score as gene ratio), color indicates significance level (score as -log_10_ (qvalue)) using Benjamini & Hochberg (BH) correction. C. Venn diagram indicates intersections of DEGs with different expression patterns in stamen organ and cell types. D. GO enrichment analysis of DEGs with different expression patterns, x-axis indicates percentage of identified genes in background (score as gene ratio), y-axis indicates enriched GO items, size indicates counts of identified genes in this item, color indicates significance level (score as -log_10_ (qvalue)) using Benjamini & Hochberg (BH) correction.


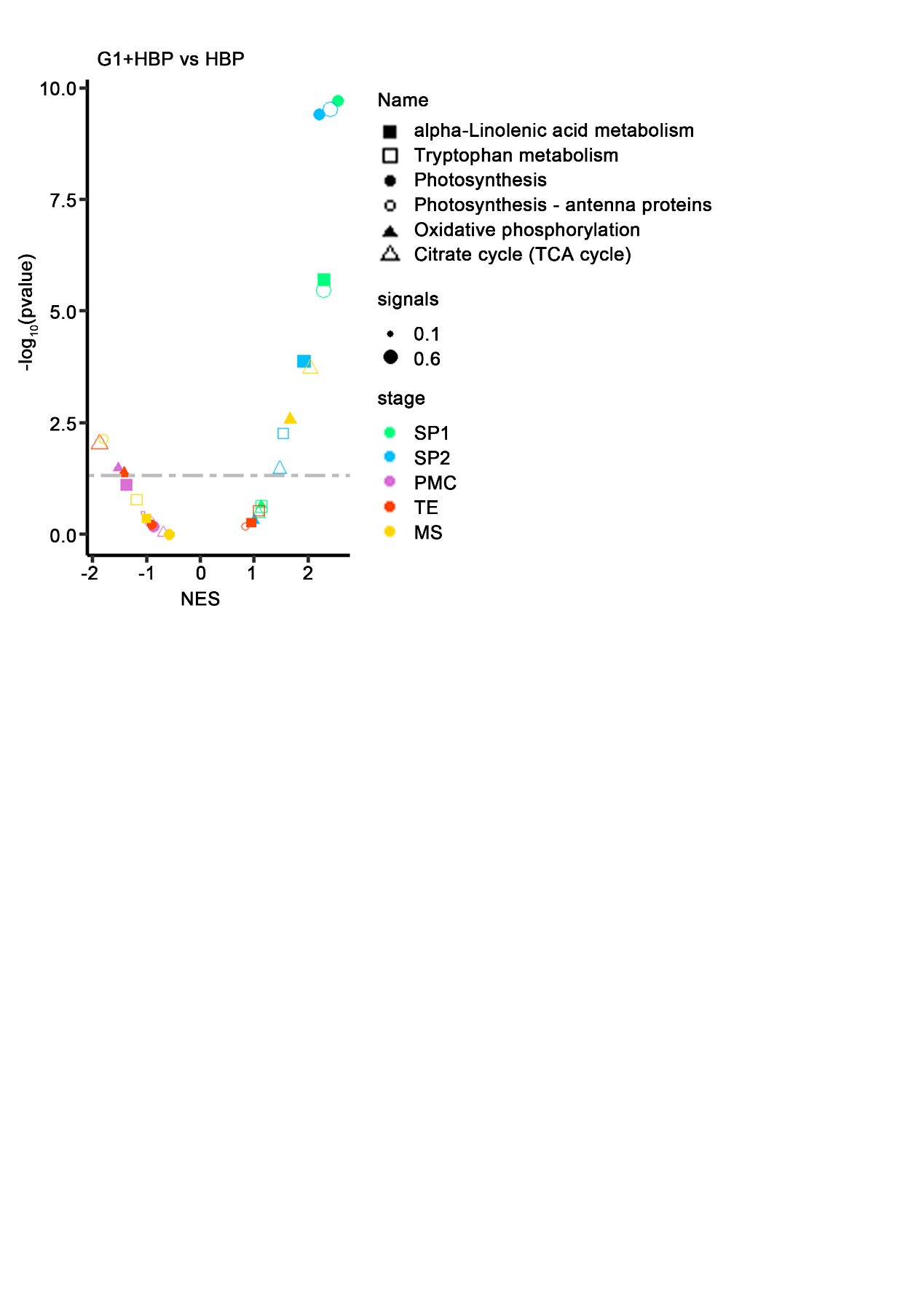


**Supplemental** **Figure S7. Significant pathways from the pairwise GSEA comparisons in stamen organ and cell types of HBP and G1+HBP.** The size of each bubble represents the signals within the pathway. The shape of each bubble represents pathway names. The color of each bubble represents GSEA comparisons of different stamen organ and cell types. NES, Normalised Enrichment Score.


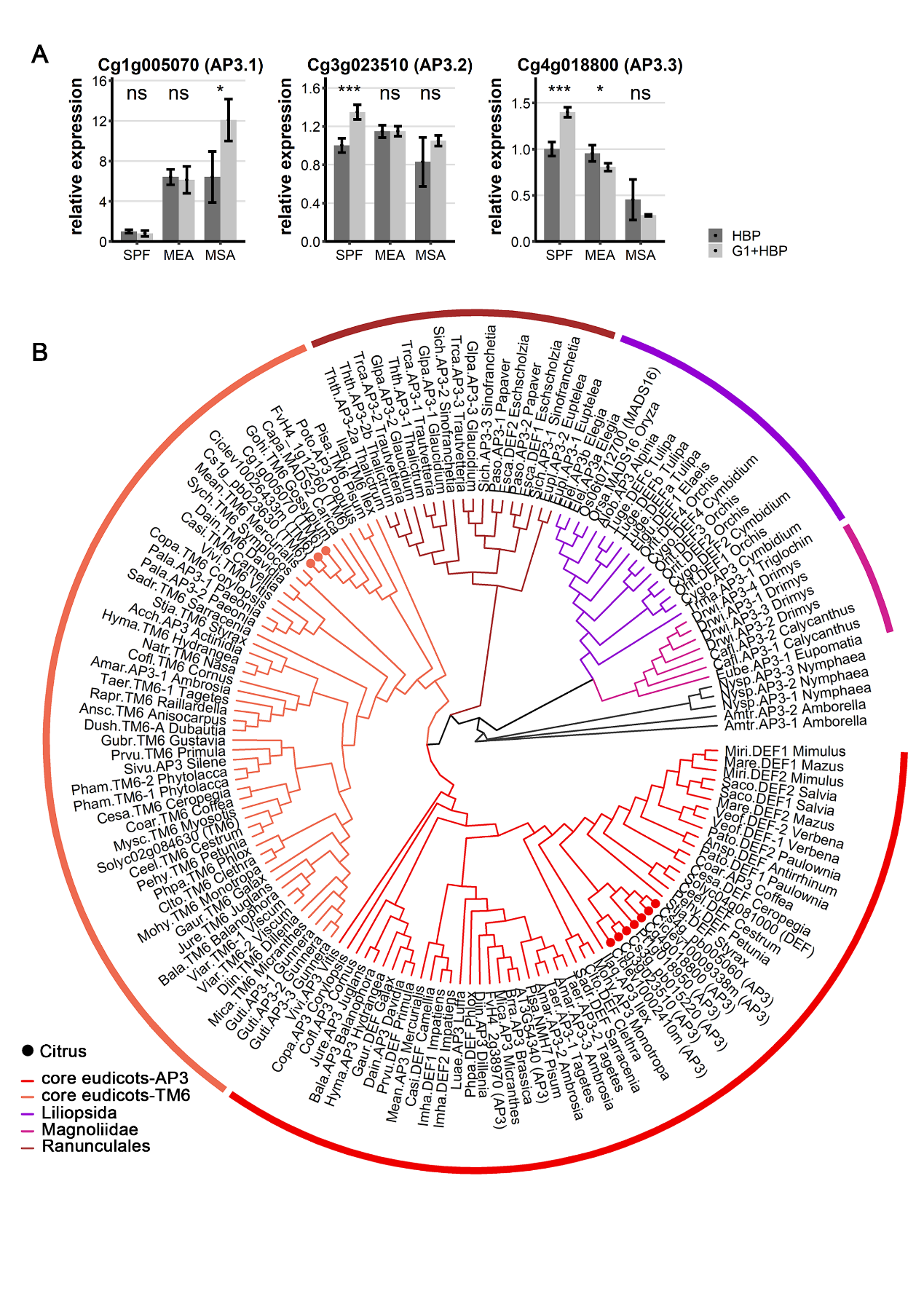


**Supplemental** **Figure S8. Relative expression of CgAP3 and Phylogenetic tree of AP3.** A. Relative expression of *CgAP3* in flower buds and anthers of HBP and G1+HBP. The gene expression differences were compared using qRT-PCR in flower bud at stamen primordia developmtn stage (SPF), anthers at meiosis stage (MEA) and anthers at microspore development stage (MSA) stage. Data are shown as means ± SD. Three biological repeats were performed, and significant differences were determined using student’s t test, * P<0.05, ** P<0.01, *** P<0.001, **** P<0.0001. B. Phylogenetic tree of representative AP3 lineage proteins from basal angiosperm, monocot, and core eudicots. Maximum likelihood analysis is conducted using iqtree.


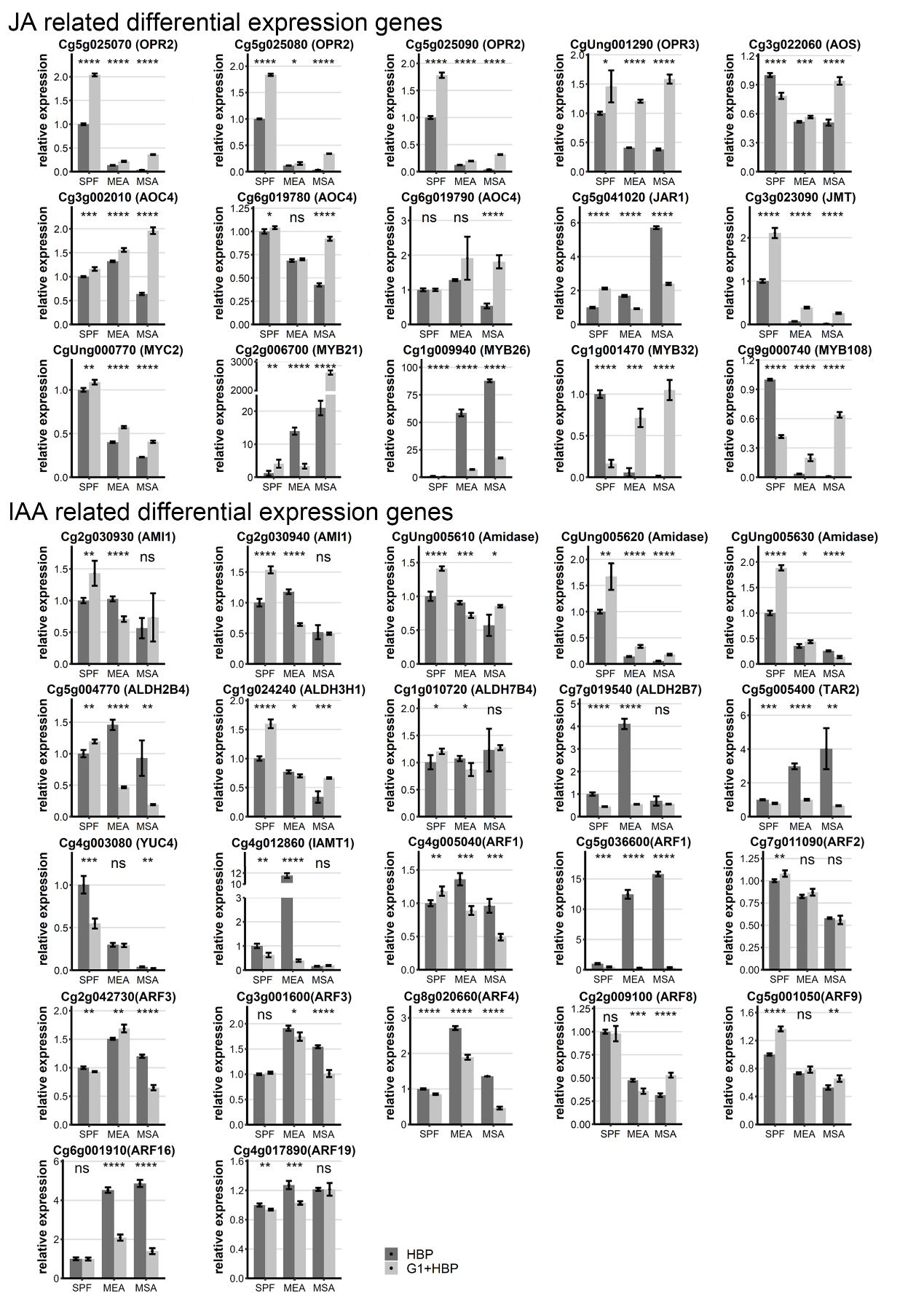


**Supplemental** **Figure S9. Relative expression level of jasmonic acid and auxin biosynthesis and signal transduction genes**. The gene expression differences were compared using qRT-PCR in flower bud at stamen primordia development stage (SPF), anthers at meiosis stage (MEA) and anthers at microspores development stage (MSA). Data are shown as means ± SD. Three biological repeats were performed, and significant differences were determined using student’s t test, * P<0.05, ** P<0.01, *** P<0.001, **** P<0.0001.


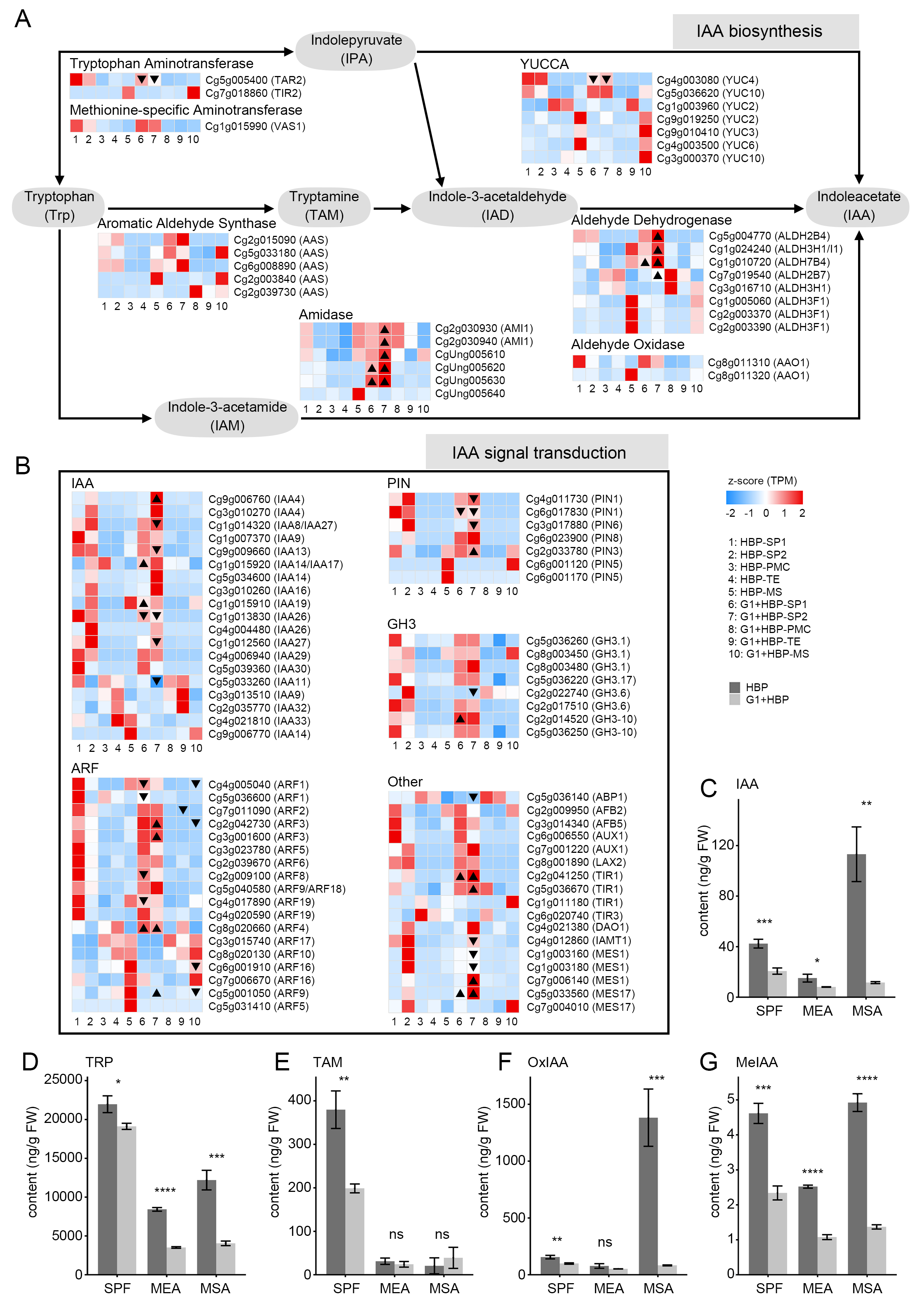


**Supplemental** **Figure S10. Expression level of auxin (IAA) pathway genes and content of auxin and its metabolites in the stamen of HBP and G1+HBP.** A. Expression level of auxin biosynthesis pathway genes in stamen organ and cell types of HBP and G1+HBP. B. Expression level of auxin signal transduction genes in stamen organ and cell types of HBP and G1+HBP. In A-B, color in heatmap indicates normalized TPM using z-score, DEGs are marked with triangles, regular triangle indicated significantly upregulated genes, inverted triangle indicated significantly downregulated genes. C-G. Content of auxin and its metabolites in flower bud with stamen primordia (SP), anthers at meiosis (ME) and microspores (MS) stages. Indole-3-acetic acid (IAA) content (C), Tryptophan (TRP) content (D), Tryptamine (TAM) content (E), 2-oxindole-3-acetic acid (OxIAA) content (F), Methyl indole-3-acetate (MEIAA) content (G). In C-G, data are shown as means ± SD. Three biological repeats were performed, and significant differences were determined using student’s t test, * P<0.05, ** P<0.01, *** P<0.001, **** P<0.0001.


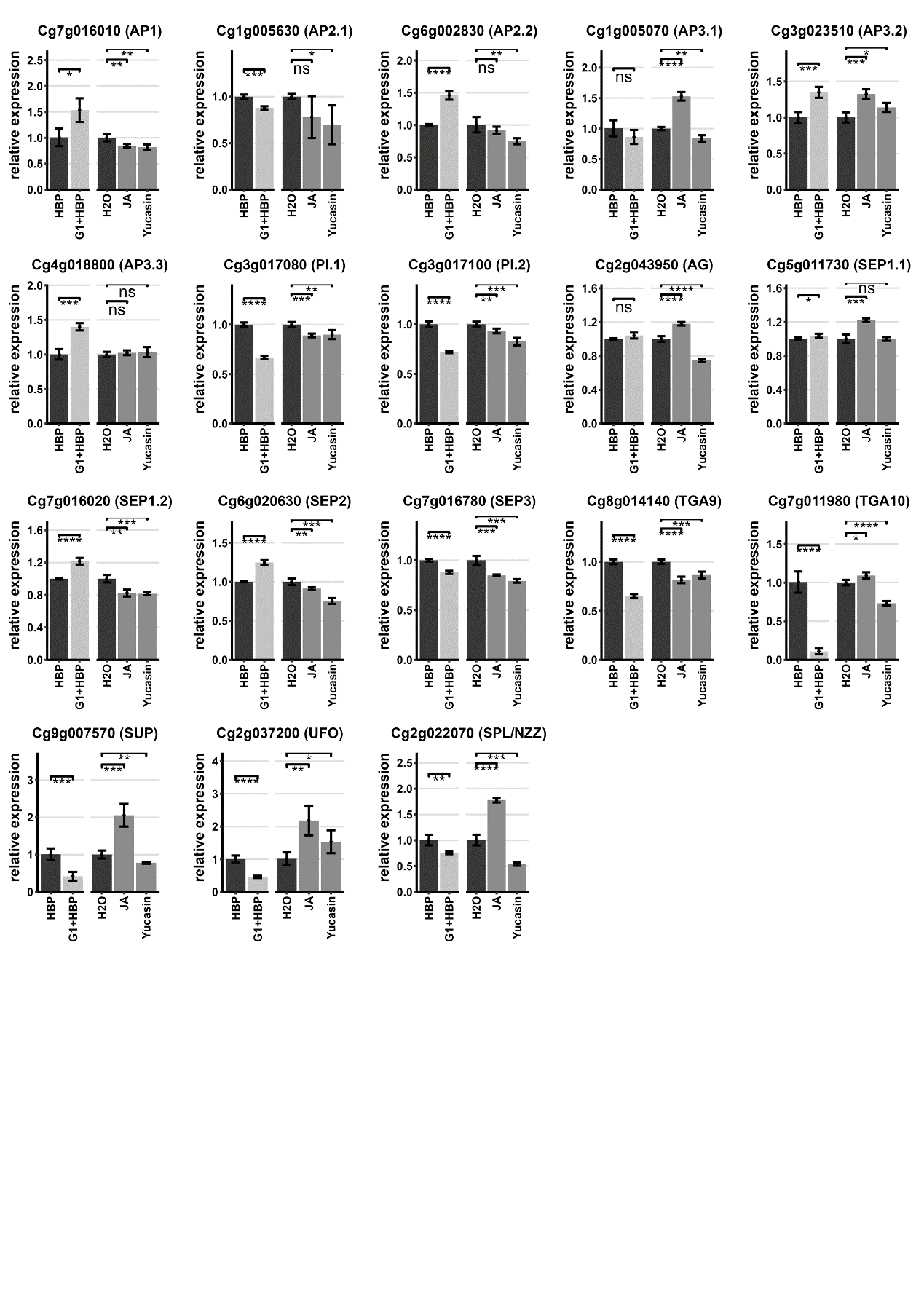


**Supplemental** **Figure S11. Relative expression level of flower development related genes**. The gene expression differences were compared using qRT-PCR in flower bud of HBP and G1+HBP, and also in flower bud after exogenous application of jasmonic acid (JA) and yucasin. Data are shown as means ± SD. Three biological repeats were performed, and significant differences were determined using student’s t test, * P<0.05, ** P<0.01, *** P<0.001, **** P<0.0001.


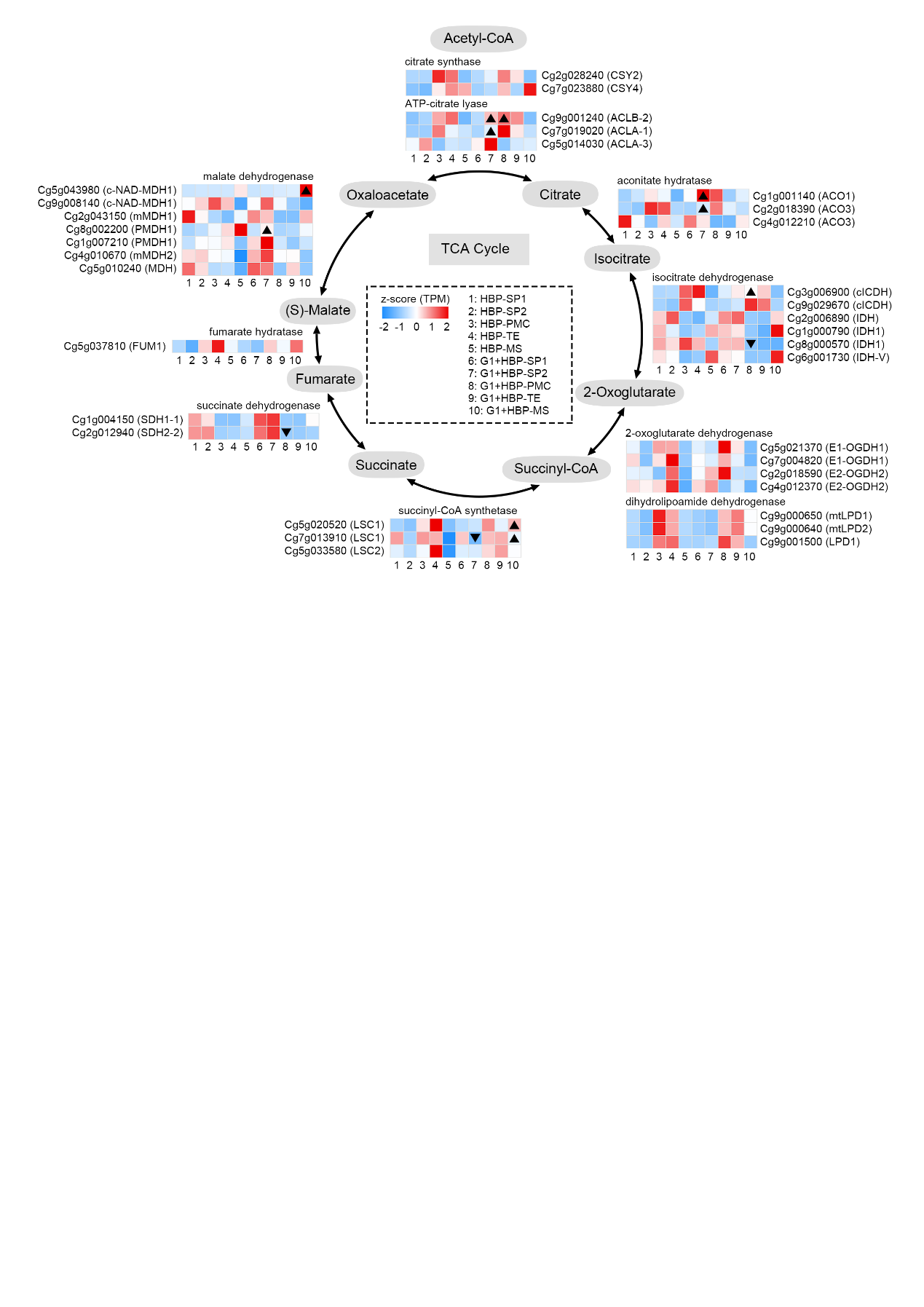


**Supplemental** **Figure S12. Expression level of tricarboxylic acid cycle (TCA) pathway genes in stamen organ and cell types of HBP and G1+HBP.** Color in heatmap indicates normalized TPM using z-score, DEGs are marked with triangles, regular triangle indicated significantly upregulated genes, inverted triangle indicated significantly downregulated genes.
